# Peptidoglycan-Modifying Enzyme Pgp1 Is Required for Helical Cell Shape and Pathogenicity Traits in *Campylobacter jejuni*

**DOI:** 10.1371/journal.ppat.1002602

**Published:** 2012-03-22

**Authors:** Emilisa Frirdich, Jacob Biboy, Calvin Adams, Jooeun Lee, Jeremy Ellermeier, Lindsay Davis Gielda, Victor J. DiRita, Stephen E. Girardin, Waldemar Vollmer, Erin C. Gaynor

**Affiliations:** 1 Department of Microbiology and Immunology, University of British Columbia, Vancouver, British Columbia, Canada; 2 The Centre for Bacterial Cell Biology, Institute for Cell and Molecular Biosciences, Newcastle University, Newcastle upon Tyne, United Kingdom; 3 Wine Research Centre, Faculty of Land and Food Systems, University of British Columbia, Vancouver, British Columbia, Canada; 4 Department of Laboratory Medicine and Pathobiology, University of Toronto, Toronto, Ontario, Canada; 5 Department of Microbiology and Immunology & Unit for Laboratory Animal Medicine, University of Michigan Medical School, Ann Arbor, Michigan, United States of America; University of Illinois, United States of America

## Abstract

The impact of bacterial morphology on virulence and transmission attributes of pathogens is poorly understood. The prevalent enteric pathogen *Campylobacter jejuni* displays a helical shape postulated as important for colonization and host interactions. However, this had not previously been demonstrated experimentally. *C. jejuni* is thus a good organism for exploring the role of factors modulating helical morphology on pathogenesis. We identified an uncharacterized gene, designated *pgp1* (peptidoglycan peptidase 1), in a calcofluor white-based screen to explore cell envelope properties important for *C. jejuni* virulence and stress survival. Bioinformatics showed that Pgp1 is conserved primarily in curved and helical bacteria. Deletion of *pgp1* resulted in a striking, rod-shaped morphology, making *pgp1* the first *C. jejuni* gene shown to be involved in maintenance of *C. jejuni* cell shape. Pgp1 contributes to key pathogenic and cell envelope phenotypes. In comparison to wild type, the rod-shaped *pgp1* mutant was deficient in chick colonization by over three orders of magnitude and elicited enhanced secretion of the chemokine IL-8 in epithelial cell infections. Both the *pgp1* mutant and a *pgp1* overexpressing strain – which similarly produced straight or kinked cells – exhibited biofilm and motility defects. Detailed peptidoglycan analyses via HPLC and mass spectrometry, as well as Pgp1 enzyme assays, confirmed Pgp1 as a novel peptidoglycan DL-carboxypeptidase cleaving monomeric tripeptides to dipeptides. Peptidoglycan from the *pgp1* mutant activated the host cell receptor Nod1 to a greater extent than did that of wild type. This work provides the first link between a *C. jejuni* gene and morphology, peptidoglycan biosynthesis, and key host- and transmission-related characteristics.

## Author Summary

Bacterial cell shape is dictated by the composition of the cell envelope component peptidoglycan. Some important pathogens have a characteristic helical corkscrew morphology that may help them burrow into mucus overlaying cells to initiate colonization and pathogenicity. One example is *Campylobacter jejuni*, the leading cause of bacterial-induced diarrheal disease in the developed world. Direct evidence supporting the hypothesis that *C. jejuni* shape is related to its pathogenicity traits has not previously been provided. We identified a gene encoding a peptidase modifying peptidoglycan that is essential for maintaining the *C. jejuni* corkscrew shape. We can now connect a *C. jejuni* gene with morphology and peptidoglycan biosynthesis. Loss of this gene was also found to affect pathogenic attributes such as chicken colonization, biofilms, motility, and activation of host inflammatory mediators. In addition, this is the first study to thoroughly characterize *C. jejuni* peptidoglycan structure and to identify a gene involved in peptidoglycan maintenance. Our findings highlight an emerging theme in bacterial pathogenesis research: the connection between bacterial cell biology and pathogenesis. Finally, our characterization of *C. jejuni* cell shape and peptidoglycan provides a starting point for further work in this area in *C. jejuni* and other bacteria with curved and helical morphologies.

## Introduction

*Campylobacter jejuni* is a helical, highly motile, Gram-negative ε-Proteobacterium and a prevalent zoonotic organism existing asymptomatically in the intestinal tract of birds and other animal species [1]–[3]. However, ingestion of as few as 500 bacteria can result in human disease [4]. *C. jejuni* is the leading cause of foodborne gastroenteritis in the developed world, causing an acute self-limiting infection of varying severity that can give rise to severe complications such as inflammatory bowel disease, reactive arthritis, and Guillain-Barré syndrome (GBS) [5].

*C. jejuni* lacks many of the frequently identified virulence factors encoded by other enteric pathogens such as pili, enterotoxins, and specialized secretion mechanisms [6], [7]. Genes affecting fundamental aspects of *C. jejuni* biology in hosts other than humans, such as stress survival, transmission, and asymptomatic colonization, also affect virulence in disease models. We found that *C. jejuni* strain 81-176 binds calcofluor white (CFW), a compound that reacts with β1–3 and β1–4 carbohydrate linkages and fluoresces under long wave UV light [8], [9]. The carbohydrate responsible for CFW reactivity in *C. jejuni* has not yet been identified, although it was previously shown not to be one of the well-characterized surface polysaccharides expressed by *C. jejuni*: the capsular polysaccharide, lipooligosaccharide (LOS), N-linked glycoproteins, or O-linked flagellar glycoproteins [10]. *C. jejuni* mutants with altered CFW reactivity can be readily identified in screens. All *C. jejuni* CFW hyper- or hyporeactive mutants characterized to date exhibit changes in pathogenesis, virulence, fundamental, and/or stress survival phenotypes ([10], [11]; E. Frirdich and E. C. Gaynor, unpublished). For instance, CFW hyper-reactive mutants overproduce biofilms, while hypo-reactive mutants are biofilm-defective. Other attributes associated with altered CFW reactivity have included defects in colonization, host cell interactions, cell envelope components, and stress survival. A CFW hypofluorescent mutant with a lesion in a novel peptidoglycan peptidase gene (*pgp1*) serves as the basis of this study.

The helical shape of *C. jejuni* has long been postulated to be critical for pathogenic attributes such as the ability to burrow through the mucosal layer during infection of zoonotic and human hosts. However, genetic components involved in modulating *C. jejuni* morphology had not previously been identified. Morphology is maintained in most bacteria by the peptidoglycan (PG) layer [12]. PG is composed of glycan chains consisting of β1–4 linked N-acetylglucosamine (GlcNAc) and N-acetylmuramic acid (MurNAc) residues that are cross-linked by short peptides. PG synthesis is a highly regulated process [13], [14] taking place at the bacterial inner membrane, where lipid II precursors are polymerized by glycosyltransferase and DD-transpeptidase reactions. In addition to the synthetic enzymes, PG hydrolases are required to cleave bonds in PG or PG fragments for insertion of nascent PG into the mature layer, regulation of cell wall growth, cell separation, PG turnover and recycling, cell lysis, and the release of PG fragments in host-pathogen interactions [15]. Almost every glycosidic and amide bond in PG can be cleaved by one or more specific PG hydrolases, and this redundancy makes it difficult to assign a specific function to a hydrolase [15], [16]. It has been speculated that murein synthases and hydrolases are part of a multienzyme complex for PG assembly [16], and several interactions between PG enzymes have been reported [14].

*Campylobacter* PG contains *meso*-diaminopimelic acid (*meso*-Dap) in its peptide side chains and is modified by O-acetylation [17], [18], but the detailed muropeptide composition had not previously been elucidated. Bioinformatic analyses identified 3 putative PG synthases in *C. jejuni*: the penicillin-binding proteins (PBPs) PBP1A (cjj81176_0536), PBP (cjj81176_0550), and PBP2 (cjj81176_0680), but there are no predicted homologs of low-molecular weight PBPs such as DD-endo- or carboxypeptidases. *C. jejuni* does have homologs of the Csd1 and CcmA endopeptidases and of the Csd3/HdpA endo/carboxypeptidase discovered recently in *Helicobacter pylori* [19], [20]. Deletion of each of these genes in *H. pylori* resulted in curved-rod morphologies with reduced levels of PG cross-linking [19], but preliminary evidence indicates that these enzymes may have a different function in *C. jejuni* (E. Frirdich and E. C. Gaynor, unpublished).

Here, we report the first *C. jejuni* gene, *pgp1*, to be identified with a role in helical morphology. Structural PG analyses of *C. jejuni* wild-type 81-176 and Δ*pgp1* mutant strains showed striking alterations in the mutant, with enzyme assays confirming Pgp1 as a novel DL-carboxypeptidase. Furthermore, using the Δ*pgp1* mutant with a straight morphology, the importance of *C. jejuni* helical shape and PG in pathogenesis attributes was examined; we identified roles in motility, biofilm formation, chick colonization, and stimulation of host cell proinflammatory mediators Nod1 and IL-8.

## Results

### CFW screen for hypofluorescent mutants identifies the *C. jejuni* 81-176 gene *pgp1*

**Figure 1:**
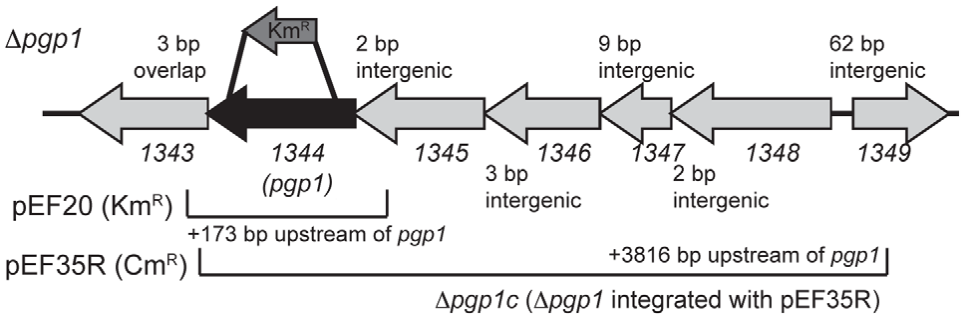
*C. jejuni* 81-176 *pgp1* gene locus. Δ*pgp1* was constructed by deleting 880 bp of *pgp1*and inserting the non-polar *aphA-3* Km^R^ cassette; the approximate location of this insertion is shown above the gene cluster and is denoted by the Δ*pgp1* strain designation. The regions cloned into the integrative vectors pRRC (pEF35R; Cm^R^) or pRRK (pEF20; Km^R^) used for complementation and overexpression, respectively, are shown below the gene cluster. An R after the plasmid name indicates that the region is cloned in the opposite direction as the antibiotic resistance cassette promoter.

As noted, screening for *C. jejuni* hypofluorescent mutants was previously shown to identify genes involved in pathogenesis-related phenotypes [10]. However, as the Tn7 transposon (Tn) used previously for mutant library construction was reproducibly found to have inserted in intergenic regions (M. K. McLennan and E. C. Gaynor, unpublished), a new random Tn library was generated as part of this study, using the highly efficient mariner system of *in vitro* Tn mutagenesis developed for *C. jejuni* [21]. The mariner library was plated onto CFW-containing plates and, from approximately 10, 000 colonies screened, 400 hypofluorescent mutants were isolated. Of the Tn insertions mapped, 8 were in distinct regions of the gene *cjj81176_1344* (Figure 1; the CFW hypofluorescent phenotype is described below). The *1344* gene was named *pgp1* (peptidoglycan peptidase 1) to describe its function and identification as the first *C. jejuni* PG peptidase to be characterized.

The *pgp1* gene product is highly conserved in mainly helical and vibrioid bacteria, primarily within the ε- and δ-Proteobacteria but also in a few extremophiles outside the ε-Proteobacteria (Table S1). The *H. pylori* homolog is described by Sycuro et al. [22]. Pgp1 was annotated as a putative periplasmic protein. However, bioinformatics using conserved domain searches and the threading program PHYRE identified an N-terminal signal peptide, a zinc binding site, and metallocarboxypeptidase catalytic residues and folds. Pgp1 contains a conserved domain at its N-terminus similar to that of the M14 family of metallocarboxypeptidases.

### Non-polar insertional deletion of *pgp1* and complementation of a *pgp1* mutant

To explore functional consequences of loss of Pgp1 function in *C. jejuni*, a non-polar *pgp1* targeted deletion strain was constructed (designated Δ*pgp1* above the gene cluster in Figure 1). To verify lack of polar effects on the downstream gene, an insertional mutation was also created in *1343*. The Δ*1343* mutant exhibited wild-type phenotypes for shape, motility, biofilm formation and CFW reactivity (data not shown; these phenotypes for Δ*pgp1* are described below). The level of *1343* mRNA was also determined to be identical in Δ*pgp1* and in the wild-type strains by semi-quantitative RT-PCR (data not shown)

The *pgp1* gene is located in the middle of a putative operon, and the location of the *pgp1* promoter is unclear from sequence analysis (Figure 1). Therefore, complementation was first attempted by expressing the *pgp1* gene from the *cat* promoter of the vector pRRC (Cm^R^) [23] integrated into an rRNA spacer region of Δ*pgp1*. This construct contained a similar region of *pgp1* as shown for pEF20 in Figure 1 (pEF20 will be described further below) and did not complement Δ*pgp1* (data not shown). However, integration of this same construct into the *C. jejuni* wild type strain also resulted in morphological and phenotypic alterations (described below), suggesting that *pgp1* copy number was likely important for complementation, and that the level of *pgp1* expression from the *cat* promoter was higher than optimal. To generate a complementing strain expressing *pgp1* from its native promoter, increasing amounts of the region upstream of *pgp1* were cloned with *pgp1* into pRRC. These regions were cloned in the opposite direction as *cat* (to avoid overexpression from the *cat* promoter) and integrated into an rRNA spacer region of Δ*pgp1*. These constructs yielded varying degrees of complementation, with the pEF35R (Figure 1) construct complementing nearly all Δ*pgp1* phenotypes to wild-type levels. The complemented strain was verified by PCR and designated Δ*pgp1*c.

### The *pgp1* gene is required for helical morphology, and Δ*pgp1* is deficient in CFW reactivity, motility, and biofilm formation

**Figure 2:**
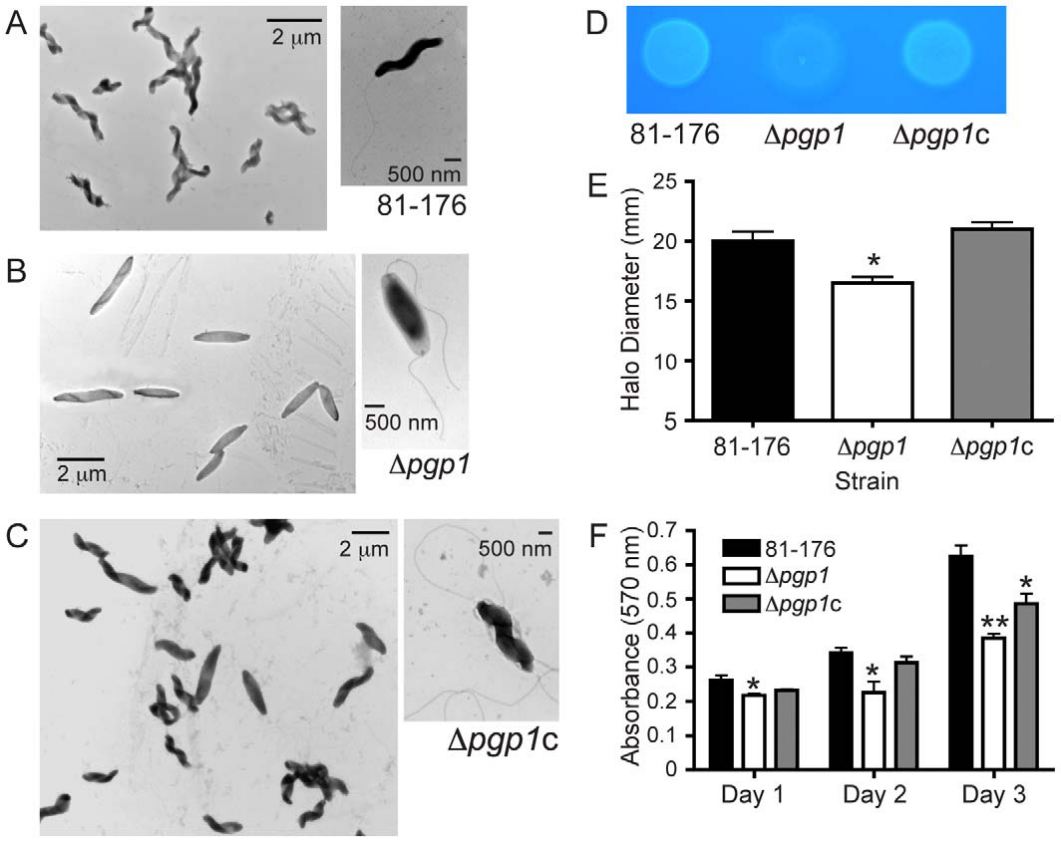
A *pgp1* mutant has a straight morphology and defects in CFW reactivity, motility, and biofilms. Negatively stained TEM images of **A,** the helical *C. jejuni* 81-176 strain; **B,** the straight Δ*pgp1* strain with intact flagella; and **C,** the complemented strain Δ*pgp1*c with restored helical morphology in 95% of the population. **D,** Δ*pgp1* is hypofluorescent after 48 h of growth on plates containing 0.002% CFW which is restored by complementation. **E,** Δ*pgp1*exhibits a slight motility defect, as assayed by measuring halo diameters in soft agar plates. Standard error of the mean was calculated from 12 measurements. **F,** Δ*pgp1* is defective for biofilm formation, partially complemented in Δ*pgp1*c. Biofilm formation was assessed by crystal violet staining of standing cultures in borosilicate tubes and quantification of dissolved crystal violet at 570 nm. Standard errors of the mean were calculated from triplicate cultures and are representative of three independent experiments. The asterisk (*) indicates a statistically significant difference using the unpaired Student's t-test, with * or ** indicating *p*<0.05 or *p*<0.01, respectively. Numerous other phenotypes showed no difference from wild type (Table S2).

Since the *pgp1* gene product is highly conserved in helical and vibrioid bacteria, the morphology of the Δ*pgp1* mutant was examined. The Δ*pgp1* mutant displayed a striking change compared to wild type and had lost the characteristic helical shape (Figure 2A), adopting a straight morphology (Figure 2B). Complementation restored the helical shape in approximately 95% of the cells (Figure 2C).

Consistent with the phenotype of the *mariner* insertion mutants, the Δ*pgp1* mutant was hyporeactive to CFW, with wild-type reactivity restored by complementation (Figure 2D). The Δ*pgp1* mutant displayed no obvious flagellar structural defects (Figure 2B), but did exhibit a slight motility defect after incubation for 20 h on 0.4% agar plates, producing halos that were, on average, about 82.5% of wild type (Figure 2E). Motility was restored to wild-type levels in the complemented strain. Biofilm levels, as assessed by a crystal violet assay, were approximately 1.2-, 1.5- and 1.6-fold lower in the Δ*pgp1* mutant compared to wild type at days 1, 2, and 3, respectively. Biofilm production was partially restored in Δ*pgp1*c (Figure 2F). No differences between wild type and Δ*pgp1* were observed for growth, stress survival, capsule and LOS migration on acrylamide gels, membrane protein composition, and sensitivity to antimicrobial compounds (Table S2).

### Pgp1 overexpression also affects helical morphology

**Figure 3:**
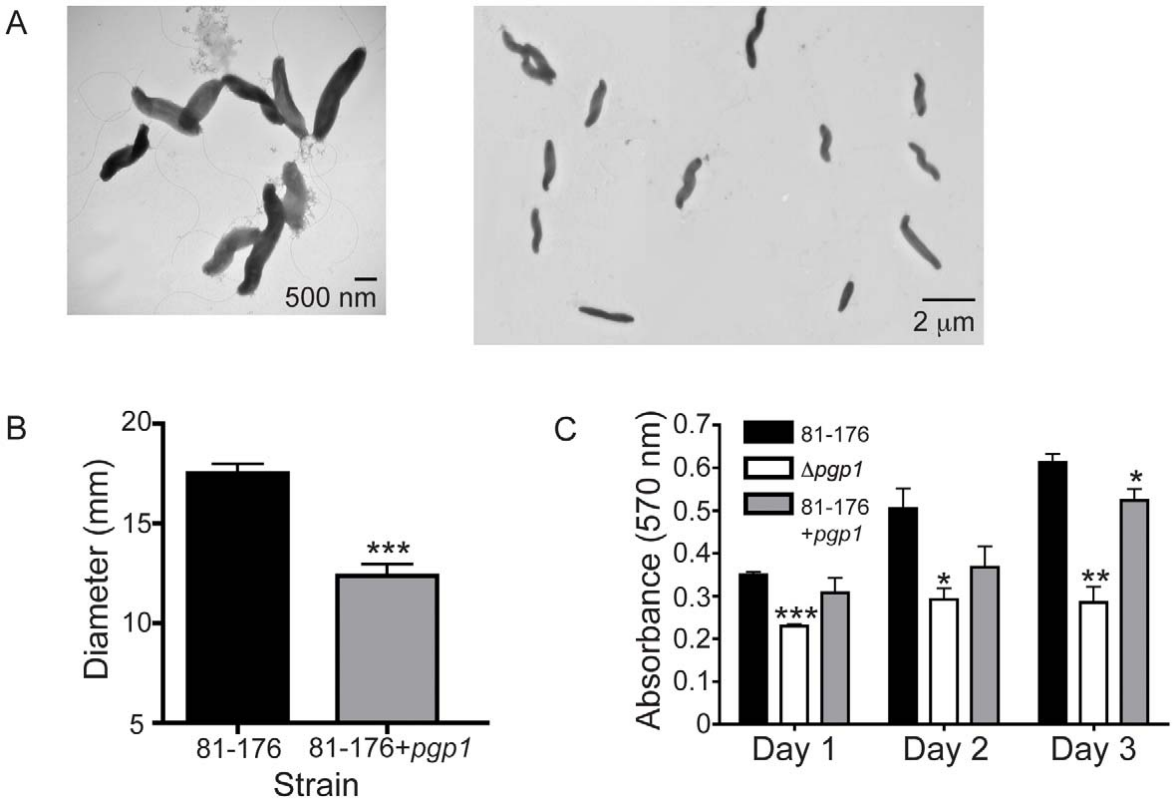
Overexpression of *pgp1* in the wild-type strain has cell straightening effects and causes reduced motility and a biofilm formation defect. The *pgp1* gene was overexpressed by integration of *pgp1* expressed from the *aphA-3* promoter from pEF20 into *C. jejuni* 81-176. **A**, negatively stained TEM images of *C. jejuni* 81-176 overexpressing *pgp1.* **B**, motility assayed by measuring halo diameter of growth in soft agar plates. Error bars were calculated from 12 measurements. **C**, biofilm formation was assessed by crystal violet staining of standing cultures in borosilicate tubes and quantification of dissolved crystal violet at 570 nm. Standard errors of the mean were calculated from triplicate readings and are representative of three independent experiments. The asterisk (*) indicates a statistically significant difference using the unpaired Student's t-test, with *, **, or *** indicating *p*<0.05, *p*<0.01 or *p*<0.001, respectively.

To assess the effects of *pgp1* overexpression, *pgp1* was expressed from either the *cat* or *aphA-3* promoter in pRRC (data not shown) or pRRK (pEF20 in Figure 1), respectively, and integrated at an rRNA spacer region of wild-type *C. jejuni*. Both pRRC and pRRK derivatives had the same effects on wild type, so only the results for pEF20 are shown. The *pgp1* overexpressing strain displayed an altered cell shape in approximately 50% of the population, producing kinked and straight cells among the helical cells (Figure 3A). This strain also exhibited reduced motility and a defect in biofilm formation (Figure 3B & C). Reverse transcriptase-quantitative PCR (RT-qPCR) confirmed that the levels of *pgp1* mRNA were 5.1-fold higher in the overexpressing strain than in wild type. In addition, there was a 1.2-fold increase in *pgp1* mRNA levels in the complementing strain Δ*pgp1*c in comparison to wild type, which may explain the partial complementation of some phenotypes. Expression of *pgp1* in *E. coli* had no effect on cell shape (data not shown).

### Peptidoglycan analysis

#### Muropeptide profile of wild-type *C. jejuni* 81-176 reveals numerous stem peptide species

**Figure 4:**
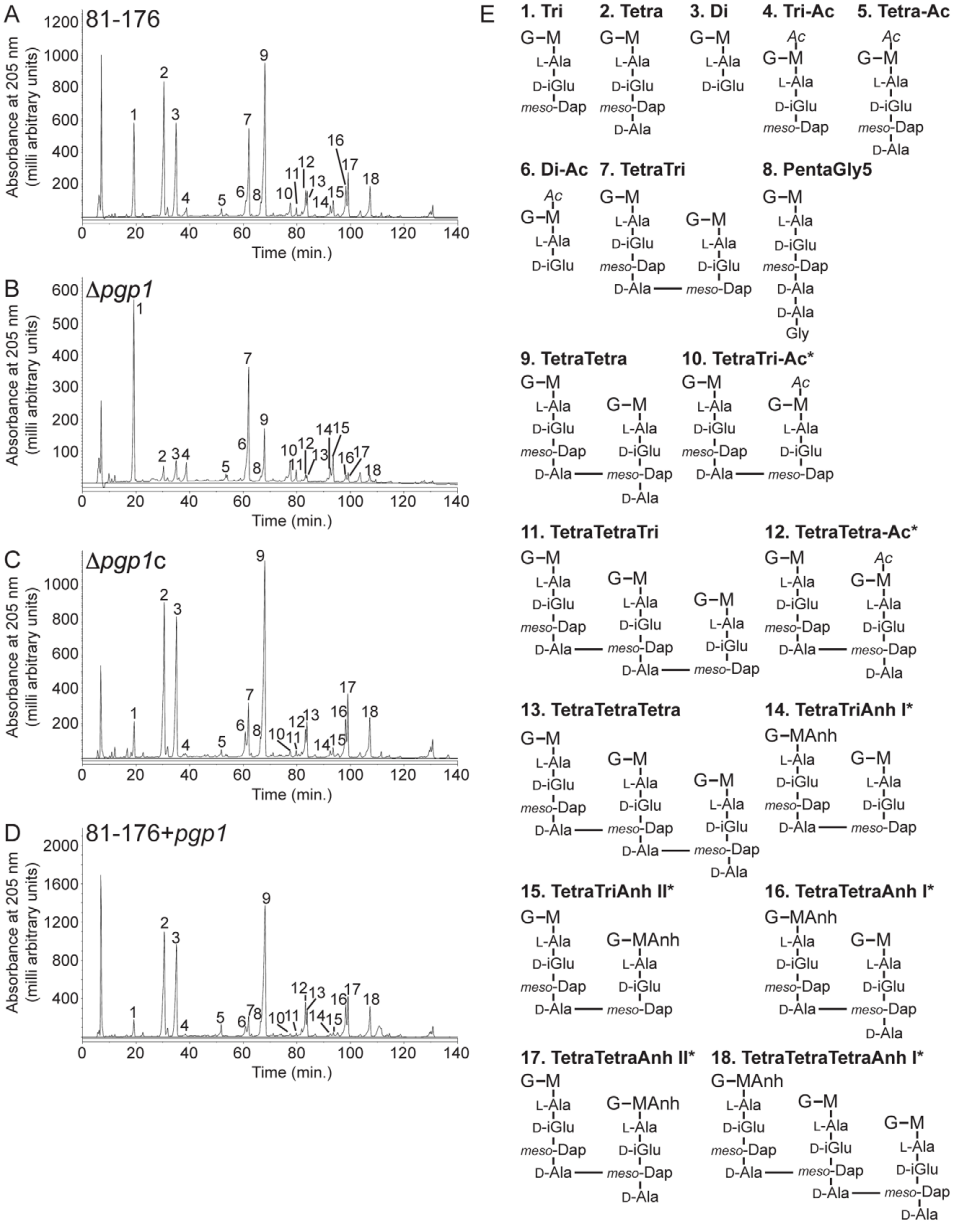
HPLC elution profile of *C. jejuni* muropeptides and proposed muropeptide structures. Purified PG was digested with cellosyl and the resulting muropeptides were reduced with sodium borohydride and separated on a Prontosil 120-3-C18 AQ reverse-phase column. HPLC profiles are shown for **A,** *C. jejuni* wild-type 81-176; **B,** Δ*pgp1*; **C,** the complement Δ*pgp1*c; **D,** the *pgp1* overexpressing strain, 81-176+*pgp1*. Peak numbers correspond to the main muropeptide peak fractions of *C. jejuni* 81-176 analyzed by LTQ-FT-MS (Table S3) to determine the structures shown in **E**. G, N-acetylglucosamine; M, reduced N-acetylmuramic acid; L-Ala, L-alanine; D-iGlu, D-isoglutamic acid; D-Glu, D-glutamic acid; *meso-*DAP, *meso-*diaminopimelic acid; Gly. Glycine; Ac, O-acetyl groups at the C-6 hydroxyl group of MurNAc; Anh, 1,6-anhydro group at MurNAc. The asterisk (*) indicates that it is not known on which MurNAc residue the modification occurs.

Given the shape phenotype of the *pgp1* mutant strains and the putative peptidase domain of Pgp1, we hypothesized that Pgp1 cleaves the PG stem peptide. Since *C. jejuni* PG had not been fully characterized, we first analyzed the muropeptide composition of wild type. The HPLC analysis is shown in Figure 4A, and the structure of individual muropeptides (Figure 4E) was confirmed by co-elution with known muropeptides from *E. coli* [24] and by LTQ-FT-MS/MS (Table 1; raw data in Tables S3 and S4) [25].

**Table 1:** Summary of muropeptide composition of *C. jejuni* wild-type 81-176, Δ*pgp1* mutant, Δ*pgp1* complement (Δ*pgp1*c), and *pgp1* overexpression (81-176+*pgp1*) strains, and the resultant Δ*pgp1* PG profiles of Pgp1 activity assays consisting of Δ*pgp1* PG incubated without enzyme, with Pgp1 in the presence of ZnCl_2_, and with Pgp1 without ZnCl_2_ but with EDTA.

	% Peak area^1^						
Muropeptide species	In *C. jejuni* strains				Following incubation of Δ*pgp1* PG		
	81-176	Δ*pgp1*	Δ*pgp1c*	81-176 + *pgp1*	Δ*pgp1* PG - Pgp1	Δ*pgp1* PG + Pgp1+ ZnCl_2_	Δ*pgp1* PG + Pgp1 + EDTA
Monomers (Total)	43.3	43.4	43.1	42.1	41.1	45.4	**52.4**
Di	14.5	5.9*	20.6*	18.4*	5.6	31.9*	5.9
Tri	10.5	34.4*	3.3*	2.3*	32.6	2.0*	33.9
Tetra	18.4	3.1*	19.2	21.5	2.9	11.5*	12.6*
Acetylated^2^	3.3	5.1	4.2	4.0	0.7	2.2	2.1
Anhydro					1.0	2.0*	3.0*
Dimers (Total)	49.7	52.5	49.3	50.0	52.4	49.0	42.3
Tetra Tri	14.7	37.0*	6.8*	3.7*	37.9	36.5	31.9
Tetra Tetra	33.5	14.7*	40.8*	44.4*	13.5	11.5	**9.6**
Tetra PentaGly^5^	1.5	0.7*	1.6	1.9*	0.9	0.9	0.8
Anhydro	10.8	11.7	11.3	10.5	12.7	7.8*	5.8*
Acetylated^2^	4.8	6.8	3.8	5.5	0.2	2.8	1.6
Trimers (total)	6.9	4.2*	7.6	7.9	6.5	5.6	5.3
Tetra Tetra Tri	0.8	1.9*	0.6*	0.3*	3.0	2.6	**2.4**
Tetra Tetra Tetra	6.1	2.3*	7.0	7.6*	3.6	3.0	2.9
Dipeptides (Total)	14.5	5.9*	20.6*	18.4*	5.6	31.9*	5.9
Tripeptides (Total)	18.1	53.6*	6.9*	4.2*	52.5	21.2*	50.7
Tetrapeptides (Total)	66.7	40.2*	71.6	76.5	41.4	46.5	43.0
Pentapeptides (Total)	0.7	0.4*	0.8	1.0*	0.5	0.5	0.4
Acetylated (Total)^2^	5.7	8.5	6.1	6.7	0.8	3.6	2.9
Anhydro chain ends (Total)	6.8	6.4	7.2	6.9	7.2	10.7*	**10.1**
Average chain length	14.7	15.7	13.9	14.5	13.9	9.3*	**9.9**
Degree of cross-linkage	29.5	29.0	29.7	30.2	30.5	28.2	24.7
% Peptides in cross-links	56.7	56.6	56.9	57.9	58.9	54.6	47.6

1 Numbers represent the percent area of each muropeptide from Table S4 calculated to give a total of 100%. Values indicated with an asterisk (*) represent an equal to or greater than 20% difference in comparison to wild-type 81-176 or Δ*pgp1* PG to which no enzyme was added; bolded asterisked values (*) indicate a greater than 30% change.

2 Values for the percentage of O-acetylated species do not represent the true level of PG O-acetylation in these strains, as most O-acetyl groups are lost in the standard alkaline muropeptide reduction procedure used in this study.

The most abundant muropeptides were the monomers with di-, tri- and tetrapeptides, as well as dimeric 4-3 cross-linked TetraTetra and TetraTri species. Only one very minor peak with a pentapeptide side chain was detected in *C. jejuni*, a TetraPenta dimer with a Gly residue at position 5, as opposed to the more common D-Ala. Muropeptides with 3-3 crosslinks or Lys-Arg residues indicating bound lipoprotein were not detected and thus are either not present or present in very small amounts. The degree of cross-linking at 29.5% is consistent with the value of 30% published previously [17]. The average glycan chain length determined by the fraction of 1,6-anhydromuramic acid containing muropeptides from chain ends was 14.7 dissacharide units. The PG glycan backbone of two different strains of *C. jejuni* was reported to be modified with O-acetyl groups at levels of 55.7% and 62% relative to MurNAc content [18]. The fact that we observed lower levels of O-acetylation in our analyses (5.7%) is likely due to the loss of most of the labile O-acetyl groups in the standard alkaline muropeptide reduction procedure applied in this study.

#### Loss of *pgp1* and Pgp1 overexpression result in altered PG muropeptide profiles

PG from the Δ*pgp1* mutant, complement (Δ*pgp1*c), and *pgp1* overexpression (81-176+*pgp1*) strains was isolated, and the muropeptide profiles were analyzed by HPLC as above (Figure 4A–D, Tables 1 and S4). The Δ*pgp1* mutant exhibited a number of changes from wild type, most significantly a striking increase in tripeptides and reduced dipeptides and tetrapeptides. The overexpression strain (81-176+*pgp1*) exhibited the opposite profile from Δ*pgp1*, with a decrease in total tripeptides, an increase in total dipeptides, and to a lesser extent, an increase in tetrapeptides and Gly-containing pentapeptides. Complementation restored many of the changes in Δ*pgp1* to near-wild-type levels; however, *pgp1* may be modestly overexpressed in Δ*pgp1*c based on some similarities to the 81-176+*pgp1* profile. The overall degree of cross-linking did not vary significantly between the different strains. Together with sequence analysis (see above), the PG data suggests Pgp1 functions as a DL-carboxypeptidase, hydrolyzing the D-Glu-*meso*-Dap bond in tripeptides to form dipeptides. Loss of most O-acetyl groups in the PG preparation techniques used in this study precluded exact quantification of this PG glycan backbone modification in the Δ*pgp1* mutant. As PG O-acetylation confers resistance to lysozyme [26], [27], it would be expected that if Pgp1 affects O-acetylation, the Δ*pgp1* mutant would display a change in lysozyme sensitivity. In the presence of the metal chelator EDTA to permeabilize the outer membrane, no difference in MIC for lysozyme was observed between wild type and Δ*pgp1* strains (Table S2). Biochemical analyses (described below) confirming Pgp1 as a DL-carboxypeptidase cleaving the PG stem peptide further suggested no functional involvement of Pgp1 in O-acetylation, thus additional experiments to assess this were not pursued.

#### Pgp1 is a metal-dependent DL-carboxypeptidase cleaving monomeric disaccharide tripeptides to dipeptides

**Figure 5:**
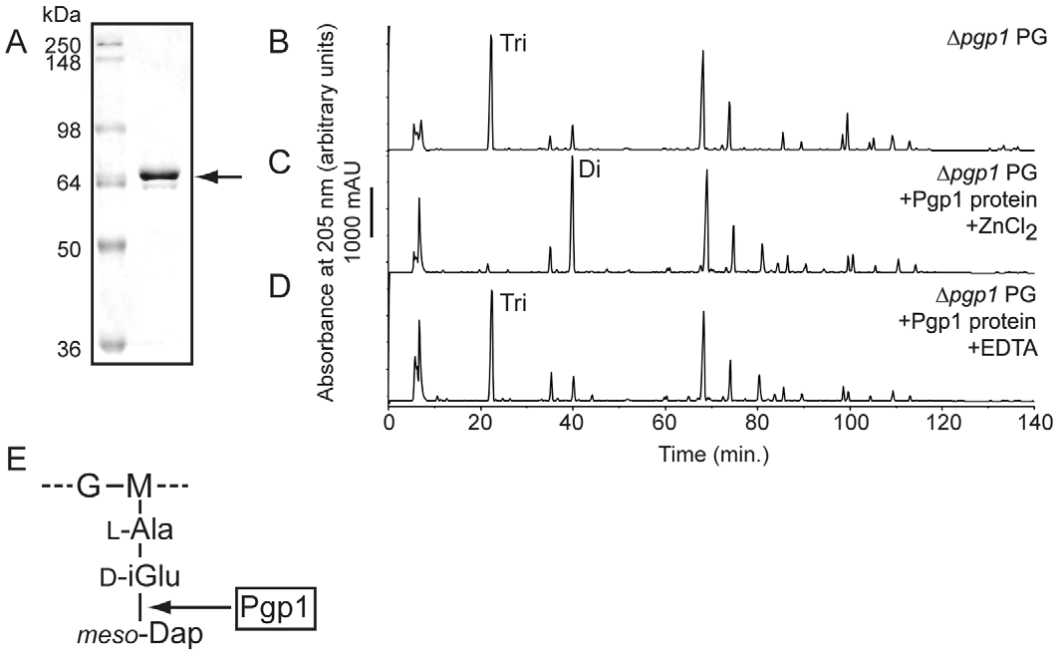
Pgp1 has metal-dependent DL-carboxypeptidase activity on Δ*pgp1* PG, cleaving monomeric tripeptide disaccharides to dipeptides. **A,** SDS-PAGE analysis of affinity purified Pgp1 with a predicted molecular weight of 67.9 kDa, indicated by an arrow. HPLC chromatograms of **B,** purified Δ*pgp1* PG; **C,** Δ*pgp1* PG incubated with purified Pgp1 and ZnCl_2_; and **D,** Δ*pgp1* PG with purified Pgp1 and EDTA. Peaks corresponding to monomeric disaccharide dipeptides and tripeptides are indicated. **E,** a schematic diagram of the Pgp1 cleavage site indicated with an arrow. G, N-acetylglucosamine; M, reduced N-acetylmuramic acid; L-Ala, L-alanine; D-iGlu, D-isoglutamic acid; *meso-*DAP, *meso-*diaminopimelic acid.

Pgp1 protein was expressed and purified from *E. coli* (Figure 5A) and incubated with purified Δ*pgp1* PG to assess the biochemical function of the protein. The resultant PG was analyzed as above, and the muropeptide profiles were compared to that of Δ*pgp1* (Figure 5B–D, Tables 1 and S4). In the presence of ZnCl_2_, the addition of Pgp1 resulted in a striking increase in monomeric dipeptides with a concomitant decrease in monomeric tripeptides, characteristic of a metal-dependent DL-carboxypeptidase (Figure 5C; Table 1). This activity was eliminated in the absence of ZnCl_2_ with added EDTA, establishing the dependence of Pgp1 on divalent ions (Figure 5D). This confirmed Pgp1 as a metal-dependent DL-carboxypeptidase cleaving monomeric disaccharide tripeptides to dipeptides (Figure 5E).

### The effect of *pgp1* on host-related phenotypes

#### *Δpgp1* is defective for *in vivo* chick colonization

**Figure 6:**
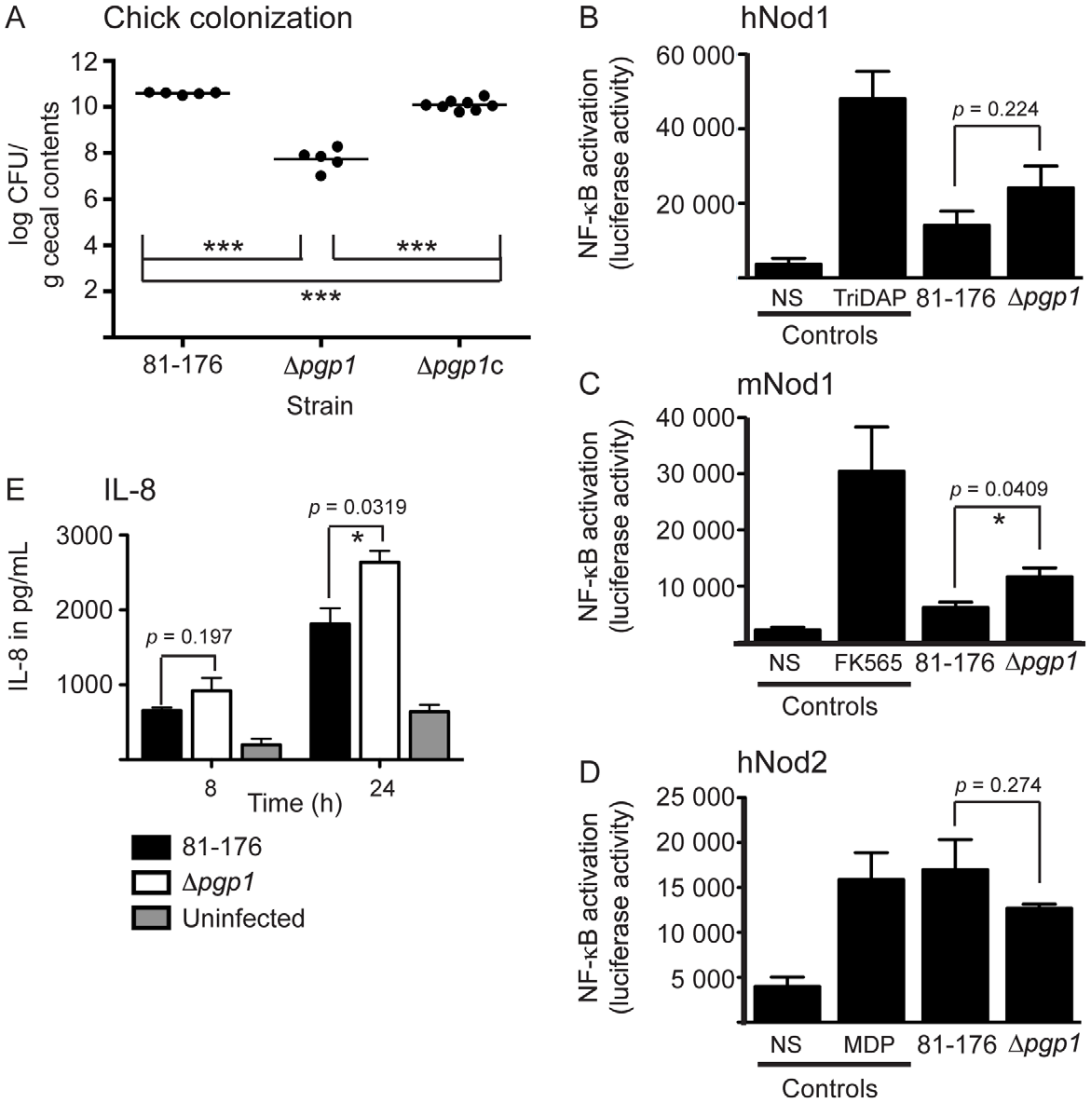
The role of cell shape and PG composition on host related phenotypes. **A,** Δ*pgp1* is defective for chick colonization. Each point represents the log CFU/g cecal contents of an individual chick 6 days following infection with 10^4^ CFUs of the indicated *C. jejuni* strains. The geometric mean is denoted by a black bar. **B–D,** to assay the ability of *C. jejuni* wild type and Δ*pgp1* PG to activate Nod proteins, human embryonic kidney cells (HEK293T) were co-transfected with either *C. jejuni* 81-176 or Δ*pgp1* PG at 0.1 µg/mL, vectors for a NF-κB luciferase reporter, and either human Nod1 (**B,** hNod1), mouse Nod1 (**C,** mNod1) or human Nod2 (**D,** hNod2). Nod activation was determined by measuring the activity of the NF-κB luciferase reporter in comparison to the non-stimulated (NS) negative control. Positive controls used were TriDAP, FK565, and MDP. Data represent the mean ± SEM of three independent experiments. **E,** Deletion of *pgp1* increases IL-8 secretion in the INT407 epithelial cell line. Quantification of IL-8 levels was performed by ELISA. Data represent the mean ± SEM of three independent experiments. The asterisk (*) indicates a statistically significant difference using the unpaired Student's t-test, with * or *** indicating *p*<0.05 or *p*<0.001, respectively.

A *C. jejuni* chick colonization model was used to determine the role of *pgp1 in vivo*. One-day old chicks were infected orally with a dose of 10^4^ *C. jejuni* wild-type 81-76, Δ*pgp1*, or Δ*pgp1*c, and cecal contents were assessed for *C. jejuni* after 6 days. The Δ*pgp1* mutant exhibited a statistically significant (*p* 0.0009) 2.9-log decrease in average levels of colonization compared to wild type (Figure 6A). Complementation restored colonization levels to within 0.5-log of wild type (Figure 6A). As a control to explore whether the slight motility defect of Δ*pgp1* may be responsible for the reduction in chick colonization, we also infected chicks with a Δ*carB* mutant [10]. *carB* encodes carbamoylphosphate synthase and is directly downstream of an intergenic Tn7 insertion in a CFW hypofluorescent mutant (*dim13*) isolated in a previous screen [10]. A Δ*carB* mutant had wild-type helical morphology, but displayed a slight motility defect of approximately 86.0% that of wild-type (Figure S1) and a hypofluorescent phenotype on CFW similar to Δ*pgp1* [10]. However, unlike Δ*pgp1*, the Δ*carB* mutant showed no statistically significant change in chick colonization (Figure S1).

#### *In vitro* invasion and intracellular survival in epithelial and macrophage cell lines are not dramatically affected in *Δpgp1*

The human epithelial cell lines T84, Caco-2 and INT407, and the murine RAW 264.7 and human Thp-1 macrophage cell lines were used in gentamicin protection assays to examine the role of *pgp1* in *C. jejuni* invasion and intracellular (IC) survival *in vitro*. No defects in invasion were observed for any cell line (2 h post gentamicin treatment). In epithelial cells, Δ*pgp1* exhibited a 0.5 log increase in IC survival compared to wild type 7 h post gentamicin treatment (shown for Caco-2 cells in Figure S2). This was also seen at 5 h and 9 h (data not shown), but not at 21 h post gentamicin treatment. No differences in IC survival were observed in either macrophage cell line tested (data not shown).

Because of the modest motility defect of Δ*pgp1* in soft agar, we also wished to assess whether attachment and invasion might be altered in a tissue culture medium of higher viscosity. MEM supplemented with carboxymethylcellulose (CMC) was previously used to demonstrate increased host cell attachment and invasion for some strains of *C. jejuni* in a higher viscosity medium [28]. INT407 cells were infected with *C. jejuni* 81-176 and Δ*pgp1* in MEM containing CMC. No significant difference in invasion was seen after 1 h or 3 h with the addition of 0.6%, 1% or 2% CMC (Figure S2). Furthermore, CMC addition decreased the total levels of invaded bacteria for both the wild type and mutant strains.

#### PG changes in *C. jejuni Δpgp1* result in an altered epithelial cell Nod1 response

PG is recognized by Nod-like receptors of the innate immune system. To test whether the PG changes resulting from a *pgp1* mutation altered Nod receptor stimulation, we measured expression of a NF-κB *lgk* luciferase reporter transfected in human embryonic kidney HEK293T cells along with either the human (h)Nod1, mouse (m)Nod1, or human (h)Nod2 receptor (Figure 6B–D). We found that PG from the Δ*pgp1* mutant produced a statistically significant increase in mNod1 activation compared to wild type. In contrast, Δ*pgp1* mutant PG did not produce a statistically significant change in stimulation of hNod1, despite a modest increase from wild type. *C. jejuni* wild type PG stimulated Nod2; however, there was no statistically significant difference in activation levels between the wild type and Δ*pgp1* mutant PG.

#### Secretion of interleukin-8 (IL-8) is increased by exposure of epithelial cells to *Δpgp1*

It was previously shown that epithelial cells produce IL-8 in response to *C. jejuni* infection [29]. To examine the effect of the Δ*pgp1* mutation on IL-8 secretion, the INT407 human epithelial cell line was infected with *C. jejuni* wild type and Δ*pgp1* strains, and the levels of IL-8 secreted into the supernatant were measured by ELISA (Figure 6E). The data shown in Figure 6E are representative of three separate experiments. IL-8 secretion from cells exposed to Δ*pgp1* after 8 h was reproducibly higher than wild type, and in some experiments was statistically significant (not shown). After 24 h, release of IL-8 in response to Δ*pgp1* was 1.5-fold higher than wild type and statistically significant in every experimental trial.

## Discussion

Bacterial cell shape programs and their impact on biology are becoming increasingly well-characterized for model organisms like *E. coli* and *Caulobacter crescentus* [30], [31]. However, studies linking bacterial shape and pathogenesis are in their infancy. This is the first report to address the morphogenesis program of the prevalent foodborne pathogen *C. jejuni*. Furthermore, apart from three annotated uncharacterized PBPs, no other *C. jejuni* PG-modifying enzymes have been identified. This is in spite of the fact that, as our study demonstrates, *C. jejuni* produces numerous muropeptide species indicative of additional unidentified and potentially novel PG hydrolases.

Our identification and characterization of *pgp1* provides the first link between cell shape and PG in *C. jejuni.* Pgp1 is conserved primarily in bacteria with curvature (Table S1). Although functions have not yet been ascribed for other putative shape-related proteins in *C. jejuni*, it is of note that helical ε- and δ-proteobacteria also typically contain the elongase and cytoskeletal components MreB, MreC, and RodA, but not MreD or RodZ. As such, unique PG and morphogenesis programs are likely to be identified both within and among these organisms, which in turn will lend new insight into their biology and pathogenesis.

Enzyme assays demonstrated that Pgp1 is a metal-dependent DL-carboxypeptidase cleaving monomeric disaccharide tripeptides to disaccharide dipeptides. This is consistent with Pgp1 harboring an N-terminal M14 metallocarboxypeptidase domain present in two other PG dipeptidyl peptidases, *E. coli* MpaA and *Bacillus sphaericus* ENP1, which cleave tri- and tetrapeptides to dipeptides [32]–[35]. This also correlates with the reduction in dipeptides and increase in tripeptides in Δ*pgp1* PG. Decreased tetrapeptides in Δ*pgp1* may be an indirect consequence of the increased level of tripeptides affecting activity of DD-carboxypeptidases, or an indirect effect on other PG hydrolases via disruption of a putative enzyme complex. Efforts to identify Pgp1 binding partners that would support the latter hypothesis are underway. Our muropeptide analysis of *C. jejuni* 81-176 wild-type PG also revealed a muropeptide composition expected for a Gram-negative species, with several interesting differences from the related helical organism *H. pylori* [19], [36] and the rod-shaped *E. coli* [14], [24] (Table S5). For instance, unlike *H. pylori*, *C. jejuni* contains very low levels of pentapeptides, suggesting high DD-carboxypeptidase activity. *C. jejuni* PG also has a higher level of cross-linking than either other species, and a relatively short average glycan chain length.

With *pgp1* being the first *C. jejuni* gene directly linked with helical morphology, it allowed for the first direct comparison between a helical wild type strain and an isogenic targeted rod-shaped mutant in critical aspects of *C. jejuni* pathogenesis and survival. The modest motility defect of the rod-shaped Δ*pgp1* mutant may in part account for the modest biofilm defect [37]–[39]. Further corroborating the *pgp1*-biofilm connection are our observations that *pgp1* is up-regulated in the hyperbiofilm-forming Δ*cprS* strain (S. Svensson and E. C. Gaynor, unpublished) and that Δ*pgp1* is hyporeactive to CFW [10]; the latter observation in turn continues to support a link between *C. jejuni* CFW reactivity and key biological and pathogenic processes. Isolated PG, which has a β1–4 disaccharide backbone, binds to CFW (E. Frirdich and E. C. Gaynor, unpublished), suggesting that changes in PG architecture in Δ*pgp1* may account for decreased CFW reactivity. However, as PG isolated from both wild type and mutant strains bound CFW equally well, a related explanation is that PG alterations affect the accessibility of CFW to binding sites on periplasmic PG molecules.

The Δ*pgp1* mutant also allowed us to explore roles for shape and PG composition on host interactions. Surprisingly, the Δ*pgp1* mutant was not defective for host cell attachment or invasion *in vitro* and even displayed a slight increase in short-term intracellular survival. Increasing the viscosity of the media did not affect the ability of the mutant to invade epithelial cells (Figure S2). It is of note that although attachment and invasion to host cells increased for some *C. jejuni* strains in higher viscosity medium [28], this was not the case for our highly invasive wild-type strain 81-176, suggesting varying invasion programs among different *C. jejuni* strains.

Intracellular *C. jejuni* was previously shown to induce host epithelial cell inflammatory responses via activation of the cytoplasmic Nod receptors [40], [41], which recognize distinct PG muropeptide molecules. The minimal molecule recognized by hNod2 is muramyl dipeptide (MDP), a structure common to PG from both Gram-negative and Gram–positive organisms [42]. hNod1 and mNod1 recognize DAP-containing muropeptides restricted to Gram-negative organisms. hNod1 exhibits a preference for tripeptides and mNod1 for tetrapeptides; however mNod1 also, to a lesser degree, recognizes DAP-containing tripeptides ([43]; J. Lee and S. Girardin, unpublished). Luciferase assays are a sensitive method to probe the capacity of a PG preparation to trigger Nod1 or Nod2 and provide complementary information to PG compositional data by HPLC and MS analyses. Using these assays, only mNod1 exhibited a statistically significant difference in activation by Δ*pgp1* PG compared to wild type. It is possible that *C. jejuni* modifies its PG in a manner that affects Nod signaling, as modifications of the PG backbone have been shown to affect how PG fragments are sensed by host cell receptors [44]. This poses an interesting question for future work. Our data also indicate that wild-type PG can activate hNod2, consistent with findings by Al-Sayeqh et al. (2010) who also showed NF-κB activation in HEK293T cells transfected with Nod2 and infected with live *C. jejuni.* While Zilbauer et al. (2007) did not observe *C. jejuni* activation of Nod2, this may reflect differences between cell lines used in these assays, and/or differences in reporter sensitivity.

Nod activation is an important aspect of innate immunity against *C. jejuni* [40], [41]; however, the mechanism by which *C. jejuni* PG fragments may reach the cytosol to activate Nod1 and/or Nod2 has not been elucidated. *C. jejuni* can be internalized into intestinal epithelial cells, surviving within an intracellular membrane-bound compartment known as the *C. jejuni*-containing vacuole (CCV) [45]. An oligopeptide transporter expressed in the early endosome has been implicated in the transport of Nod ligands from the endosome to the cytoplasm in HEK293T cells [46]; a similar system could allow cytoplasmic delivery of PG fragments from *C. jejuni* in the CCV. Another possibility is that during growth in the extracellular environment *C. jejuni* may shed part of its PG, as shown for other bacteria [44], [47], [48]; released Nod1 and Nod2-stimulatory molecules could then be transported to the cytoplasm by oligopeptide transporters or taken up by phagocytosis or clathrin-dependent endocytosis and transported to the cytosol to activate Nods [44], [49]–[51]. Bacterial outer membrane vesicles may also play a role in delivering *C. jejuni* Nod ligands to the cytoplasm [52].

Production of IL-8 and other proinflammatory mediators by intestinal epithelial cells infected with *C. jejuni* is thought to be key to the development of diarrhea and clearance of infection. Zilbauer et al. (2007) suggested that *C. jejuni* Nod1 activation is the primary signaling event required for IL-8 expression in Caco-2 intestinal epithelial cells. However, other work indicates that *C. jejuni*-induced IL-8 secretion can be triggered by other pathways in addition to the activation of Nod1, such as through Toll-like receptors (TLRs) and a pathway independent of Nods and TLRs that has yet to be identified [40], [53]–[58]. Thus while the increase in IL-8 secretion in response to Δ*pgp1* may be due to the modest increase in hNod1 activation observed in our luciferase assays, it cannot be ruled out that deletion of *pgp1* leads to a change in another, as-yet unidentified factor stimulating IL-8 expression. Future work is planned to address these hypotheses.

Our colonization data are in agreement with the longstanding hypothesis that the corkscrew morphology of *C. jejuni* is critical for burrowing into the mucus layer; however, the impact of shape and PG on colonization may be multifactorial. For instance, motility is a key factor in colonization [59]–[64], thus the Δ*pgp1* colonization defect may in part be due to its decreased motility. However, a Δ*carB* mutant with a similar motility defect was not deficient for chick colonization (Figure S1). When the strongly colonizing *C. jejuni* strain 305/94 was re-isolated from chickens, it displayed a more pronounced motility defect than Δ*pgp1* despite having normal flagella, and had lost its helical shape, exhibiting a similar straight morphology as Δ*pgp1* [65]. It is intriguing to hypothesize that not only shape, but also the underlying structure of the PG may play an important role in colonization. We have found that a straight phenotype can arise from various changes in muropeptides based on preliminary analysis of other straight mutants identified in our laboratory (E. Frirdich, J. Vermeulen, and E. C. Gaynor, unpublished). PG changes or changes resulting from the loss of *pgp1* could cause as-yet uncharacterized alterations to the cell surface affecting the ability of *C. jejuni* to survive in the chicken cecum, or affect host cell interactions and stimulation of innate immune receptors. Our understanding of the chicken innate immune system and how it responds to *C. jejuni* is in its infancy. The chicken genome encodes Nod1 but not Nod2, although chickens possess an ortholog of the NLRP3/NALP3 Nod-like receptor that is similar to Nod2 and also binds MDP [66], [67]. NLRP3 may substitute for Nod2 in chickens. Further insight into mechanisms by which *C. jejuni* survives commensally in the chicken cecum requires additional studies of these and other innate immune system factors.

Identification and characterization of *pgp1* provides a critical first step in understanding how shape and PG modifications impact *C. jejuni* pathogenesis. However, the shape program itself in *C. jejuni* is likely to be somewhat complicated. For instance, to the best of our knowledge, Pgp1 is the only PG modifying enzyme to date where overexpression causes cell straightening, indicating that the precise dose of Pgp1 is important for maintenance of morphology. One interpretation of this finding is that the proper ratio of monomeric tripeptides to dipeptides, which is disrupted in both the Δ*pgp1* mutant and *pgp1*-overexpressing strains, may be required for proper shape determination. It is also possible that excess Pgp1 could, as suggested for loss of Pgp1 above, indirectly affect other PG hydrolases by disrupting the stoichiometry of a putative PG biosynthetic/modification complex. Additional complexities in the shape program are evidenced by reports of straight *Campylobacter* strains in the literature for which the morphological change has not yet been attributed to mutations in specific gene product(s). For instance, some *C. jejuni* flagellar mutants appeared to have a straight morphology [68]–[70]; however, other mutants with lesions in the same flagellar genes remained helical ([71], [72]; E. Frirdich and E. C. Gaynor, unpublished). Two reports also cited that passage through the chicken gut or chick embryos resulted in straight *C. jejuni* isolates [65], [73]. Conversely, laboratory passage has led to the isolation of poorly colonizing rod-shaped *C. jejuni* and *C. coli* strains [74], [75]. In the 11168-GS (straight) and 11168-O (helical) strains of the 2004 study, sequence and expression levels of *pgp1* were found to be identical (E. Frirdich and E. C. Gaynor, unpublished). Collectively, this suggests not only that genes other than *pgp1* affecting morphology remain to be discovered, but also that mechanisms such as phase variation and/or epigenetics may be involved.

The *pgp1* homolog in *H. pylori* (named *csd4*) was identified in a screen for cell shape mutants and is described by Sycuro et al. [22]. Morphology, PG profile, and enzymatic activity indicate conserved functions for the gene products in both organisms. While some enzymes and their effects on morphology are conserved between these related helical bacteria, there are also likely to be differences in the overall PG remodeling and shape determining programs, particularly given their very different wild-type muropeptide profiles.

The Δ*pgp1* mutant will be a valuable tool to continue to study the effects of the loss of *C. jejuni* helical shape on its biology and pathogenesis. So far, *pgp1* has been found to be important for all aspects of the *C. jejuni* life cycle. As this study represents the first identification of a gene involved in *C. jejuni* helical shape and of a role for PG in shape determination, it will also provide the basis for work characterizing additional enzymes involved in *C. jejuni* PG biosynthesis and shape determination. These studies have now been made easier by the availability of PG structural data for the wild-type *C. jejuni* strain 81-176 published as part of this work. Future detailed biochemical and structural studies on Pgp1 will also provide interesting insight into the function of this key protein in *C. jejuni* physiology.

## Materials and Methods

### Ethics statement

Animal experiments were carried out in strict accordance with the University of Michigan Committee on Use and Care of Animals (UCUCA). Animal infection and euthanasia protocols were approved by the University of Michigan UCUCA and assigned approval number 10462. Oral gavage was carried out under humane guidelines using an approved protocol judged not to cause distress or harm to the animals. Euthanasia was carried out under humane guidelines using a lethal dose of isofluorane. All animal use procedures are in compliance with University guidelines, State and Federal regulations, and the standards of the National Institutes of Health Guide for the Care and Use of Laboratory Animals. The University of Michigan Animal Welfare Assurance Number on file with the NIH Office of Laboratory Animal Welfare (OLAW) is A3114-01, and the University is fully accredited by the Association for the Assessment and Accreditation of Laboratory Animal Care International (AAALAC, Intl.).

### Bacterial strains and growth conditions

Bacterial strains and plasmids used in this study and their construction are described in Text S1. *C. jejuni* strains were grown at 38°C in Mueller-Hinton (MH; Oxoid) broth or 8.5% (w/v) agar supplemented with vancomycin (10 µg/mL) and trimethoprim (5 µg/mL) (unless otherwise indicated) under microaerobic/capnophilic conditions (6% O_2_, 12% CO_2_) in a Sanyo tri-gas incubator for plates or using the Oxoid CampyGen system for broth cultures. Growth media were supplemented with chloramphenicol (Cm; 20 µg/mL) or kanamycin (Km; 50 µg/mL), where appropriate. *E. coli* strains used for plasmid construction were grown at 37°C in Luria-Bertani (LB; Sigma) broth or 7.5% agar (w/v) and supplemented with ampicillin (100 µg/mL), Cm (15 µg/mL), or Km (25 µg/mL), as necessary.

### RNA extraction, cDNA synthesis and RT-qPCR

RNA was extracted from log phase broth bacteria (OD 0.3), and cDNA was generated from the RNA, as described previously [74], [76]. The expression ratio of *pgp1* was determined relative to the C. jejuni *gyrA* gene encoding DNA gyrase subunit A. The primer and TaqMan probe sequences used for qPCR are described in Text S1. Each qPCR reaction mixture consisted of 25 ng of cDNA, 950 nM of each primer, 250 nM TaqMan probe, and 10 µl 2× TaqMan Gene Expression Master Mix (Applied Biosystems) in a total volume of 20 µl. Duplicate reactions for the gene of interest and the housekeeping gene were run in a Stratagene Mx3000P real-time PCR system for 2 min at 60°C, 10 min at 95°C, and then 40 cycles of 15 s at 95°C and 1 min at 60°C. The quantitative PCR cycle threshold (*C_T_*) results were analyzed by the comparative *C_T_* method (ΔΔ*C_T_* method).

### Calcofluor white (CFW) mutant screen

The *C. jejni* 81-176::*solo*(Km^R^) and *C. jejuni* 81-176::*picard*(Cm^R^) Tn libraries (Tn library construction is described in Text S1) and were screened on CFW, as described previously [10].

### Microscopy

Transmission electron microscopy (TEM) was carried out on 18 h broth cultures. Samples were fixed in a final concentration of 2.5% (v/v) of gluteraldehyde for 2–3 h on ice. Cells were then harvested, resuspended in an equal volume of H_2_O, and stored at 4°C. For imaging, 2 µL of bacteria was spotted onto parafilm to which 4 µL of 0.5% uranyl acetate was added for 1 min. A formavar-carbon film on 300 mesh copper grid (Canemco, Lakefield, Quebec, Canada) was added to the bacteria-uranyl acetate spot for 2 min. The grid was then removed, dried, washed ten times in sterile water, dried again and visualized on a Hitachi H7600 TEM equipped with a side mount AMT Advantage (1 mega-pixel) CCD camera (Hamamatsu ORCA) at the UBC Bioimaging facility (The University of British Columbia, Vancouver, BC, Canada).

### Phenotypic characterization: CFW, motility, and biofilm formation

Phenotypic assays were carried out with strains grown in shaking MH-TV broth or biphasic cultures grown for 18 h. CFW fluorescence was assayed as described previously [10]. For motility, cultures were diluted to an OD_600_ of 0.2 in MH-TV and 2 µl was point inoculated into MH-TV plates containing 0.4% agar. Plates were incubated for 20 h and the halo diameter was measured. Biofilm formation was assayed using crystal violet as described previously [11] with the exception that the absorbance was measured at 570 nm.

### Peptidoglycan isolation and muropeptide analysis

*C. jejuni* strains were passaged once from frozen stocks and then passaged to 20–25 MH plates and grown for 20 h to obtain log-phase bacteria at a final OD of 200–600. Cells were collected into cold MH broth by scraping, harvested by centrifugation at 8 000× g for 15 min and then resuspended in 6 mL ice cold H_2_O. Cells were lysed by dropwise addition to 6 mL 8% SDS boiling under reflux. PG was purified from the cell lysate, digested with the muramidase cellosyl (kindly provided by Hoechst, Frankfurt, Germany), and the resulting muropeptides were reduced with sodium borohydride and separated by HPLC as described [77]. Muropeptide fractions were collected, concentrated in a SpeedVac, acidified by 1% trifluoroacetic acid, and analysed by offline electrospray mass spectrometry on a Finnigan LTQ-FT mass spectrometer (ThermoElectron, Bremen, Germany) at the Newcastle University Pinnacle facility as described [25]. Muropeptide structures were assigned based on (i) comparison with retention times of known muropeptides from *H. pylori*, *Caulobacter crescentus* and *E. coli* and (ii) the obtained MS data (Table S3) and MS/MS fragmentation patterns (not shown).

### Expression, purification and enzymatic activity of Pgp1

The *C. jejuni* 81-176 *pgp1* gene was cloned for expression in *E. coli* without its signal peptide (amino acids 16–464 of the protein) in frame with the thioredoxin- and His-tag of the pET32a vector to give plasmid pEF46. A detailed description of the cloning of the expression construct, and the expression and purification protocol is included in Text S1. For enzyme assays, the purified protein was dialysed against 0.05 M Tris-Cl, pH 7.5 containing 0.01 M ZnCl_2_, 0.3 M NaCl and 20% glycerol. Purified Δ*pgp1* PG (1.0 mg/ml) was incubated with Trx-His_6_-Pgp1 (5 mM) in 0.02 M NaH_2_PO_4_, pH 4.8, 0.005 M ZnCl_2_, and 0.1 M NaCl for 4 h at 37°C on a Thermomixer at 750 rpm. A control sample received no enzyme and another enzyme sample contained 0.01 M EDTA and no ZnCl_2_. The samples were incubated with 10 µg of cellosyl (Hoechst, Frankfurt am Main, Germany) for 1 h, boiled for 10 min and centrifuged at room temperature for 15 min at 16 000 g. The muropeptides present in the supernatant were reduced with sodium borohydride and analyzed by HPLC, as described [24].

### Chick colonization

Chick colonization was performed as described previously [61], [76], with an infective dose of 10^4^ CFU. Chicken experiments were carried out under protocol #10462 approved by the University of Michigan Committee on Care and Use of Animals (UCUCA).

### *In vitro* invasion and intracellular survival in epithelial and macrophage cell lines

The human epithelial cell lines T84, Caco-2 and INT407 and the murine RAW 264.7 and human Thp-1 macrophage cell lines were used for *C. jejuni* infections. Carboxymethylcellulose (CMC) was added to the tissue culture media to increase viscosity. A detailed description of the tissue culture infections is included in Text S1.

### Epithelial cell responses & Nod activation assays

Luciferase asssays were performed as previously described [46]. Briefly, HEK293T cells were transfected overnight with 75 ng of NF-κB luciferase reporter plasmid (Igκ-luc, Invitrogen) and either human Nod1 (hNod1, 3 ng), mouse Nod1 (mNod1, 0.1 ng), or human Nod2 (hNod2, 0.1 ng). The empty vector (pcDNA3.1, Invitrogen) was used to balance the transfected DNA concentration. At the same time, either *C. jejuni* 81-176 or Δ*pgp1* PG muropeptides at 0.1 µg/mL were added, and the NF-κB-dependent luciferase activation was then measured following 18–24 h of co-incubation. Positive controls were TriDAP (tripeptide l-Ala-γ-d-Glu-*meso*-DAP, 5 µg/ml), FK565 (a synthetic immunostimulant tetrapeptide, heptanoyl-d-glutamyl-*meso*-DAP-d-alanine, 5 ug/ml), and MDP (muramyldipeptide, 10 µg/ml) for hNod1, mNod1, and Nod2 assays, respectively. Data are representative of three independent experiments.

### Interleukin-8 quantification

The INT407 human epithelial cell line was seeded at approximately 1×10^5^ cells/ml in MEM supplemented with 10% FBS into 24-well tissue culture plates and allowed to grow for 20–24 h prior to infection. The cells were washed three times with MEM and either left uninfected or infected with either *C. jejuni* wild-type strain 81-176 or Δ*pgp1* at an O.D._600_ of 0.002/mL taken from an 18 h shaking broth culture. Supernatants were collected after 8 h and 24 h, centrifuged for 10 min to pellet residual cells and bacteria, and frozen at −80°C until assayed. The concentration of IL-8 present in the supernatants was measured by the human IL-8 ELISA kit (Invitrogen, Camarillo, CA).

## Supporting Information

Figure S1 ***C. jejuni*** **Δ** ***carB*** **has a motility defect, but is unaffected for chick colonization.** **A,** negatively stained TEM images of Δ*carB* showing the full-length flagella and helical morphology. **B,** Δ*carB* exhibits a slight motility defect, as assayed by measuring halo diameters in soft agar plates. Standard error of the mean was calculated from 8 measurements. The asterisks (***) indicate a statistically significant difference (*p*<0.001) using the unpaired Student's t-test. **C,** Δ*carB* shows no defect for chick colonization. Each point represents the log CFU/g cecal contents of an individual chick 6 days following infection with 10^4^ CFUs of the indicated *C. jejuni* strains. The geometric mean is denoted by a black bar.
(TIF)

Figure S2 ***C. jejuni*** **Δ** ***pgp1*** **shows slightly enhanced intracellular survival, but no defect in invasion in the presence of carboxymethylcellulose (CMC).** **A,** a gentamicin (Gm) protection assay was used to assess the invasion and intracellular survival ability of Δ*pgp1* in Caco-2 intestinal epithelial cells. Gm was added 3 h post-infection with bacterial strains. After 2 h the Gm was washed off and the cells were incubated with fresh MEM containing 3% FBS and a low dose of Gm. **B,** infections were carried out in the presence of MEM containing 1% and 2% CMC to examine the ability of Δ*pgp1* to invade INT407 epithelial cells in higher viscosity media. CFUs were determined for each well by lysing the cells with water and plating the dilutions onto MH-TV plates. Standard errors of the mean were calculated from triplicate readings and are representative of three independent experiments. The asterisk (*) indicates a statistically significant difference (*p*<0.05) using the unpaired Student's t-test.
(TIF)

Table S1 **BLAST results for the** ***C. jejuni*** **81-176** ***1344/pgp1*** **gene product.**
(DOC)

Table S2 **Summary of Δ** ***pgp1*** **phenotypes tested that were similar to wild type.**
(DOC)

Table S3 **Molecular mass of** ***C. jejuni*** **81-176 muropeptides (reduced form) separated by HPLC and analyzed by LTQ-FT-MS.**
(DOC)

Table S4 **Muropeptide compositional analysis used to determine the values in** **Table 1** **.**
(DOC)

Table S5 **PG muropeptide composition of** ***C. jejuni*** **wild-type strain 81-176 in comparison to** ***H. pylori*** **and** ***E. coli*** **.**
(DOC)

Text S1 **Supplemental materials and methods.**
(DOC)
