# Correction: Peptidoglycan-Modifying Enzyme Pgp1 Is Required for Helical Cell Shape and Pathogenicity Traits in *Campylobacter jejuni*

**DOI:** 10.1371/annotation/aac1c73d-b014-4cee-8dbc-eb8472de211d

**Published:** 2012-10-02

**Authors:** Emilisa Frirdich, Jacob Biboy, Calvin Adams, Jooeun Lee, Jeremy Ellermeier, Lindsay Davis Gielda, Victor J. DiRita, Stephen E. Girardin, Waldemar Vollmer, Erin C. Gaynor

There are six changes that need to be made, including two changes to figures and four changes to the body text.

Corrected figures:

1. There was an extra D-Ala in Structure 8 of Figure 4E. Please see the corrected Figure 4 here:

**Figure:**
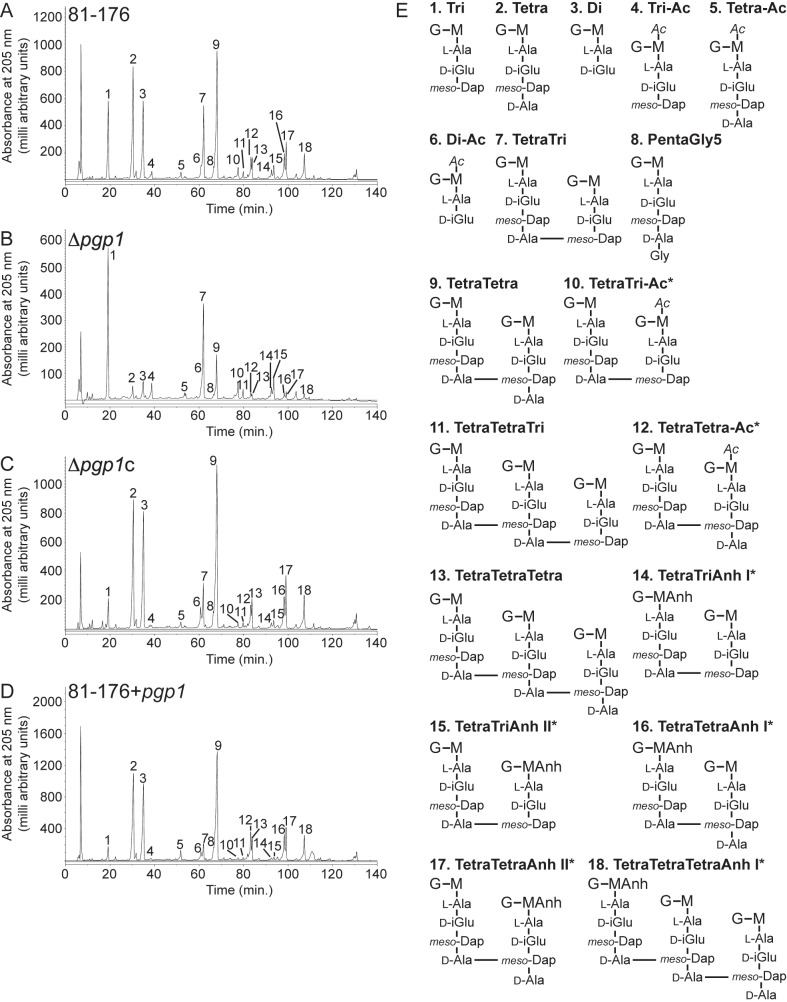


2. By using a more appropriate method of statistical analysis (paired instead of unpaired t-test), the differences in activation of hNod1 by the mutant strain become statistically significant (consistent with the PG and IL-8 analyses presented) and activation of mNod1 non-significant, while the opposite is stated in the published paper. The paired t-test is a more accurate method of analyzing the Nod activation data, as each set of data for the wild type and mutant come from an experiment carried out in duplicate on different days with each experiment exhibiting different levels of control activation. This changes the p values stated in Figure 6 from 0.224 to 0.0452 in 6B (which as noted becomes statistically significant), from 0.0409 to 0.133 in 6C, and from 0.274 to 0.293 in 6D. Please see the corrected Figure 6 here:

**Figure:**
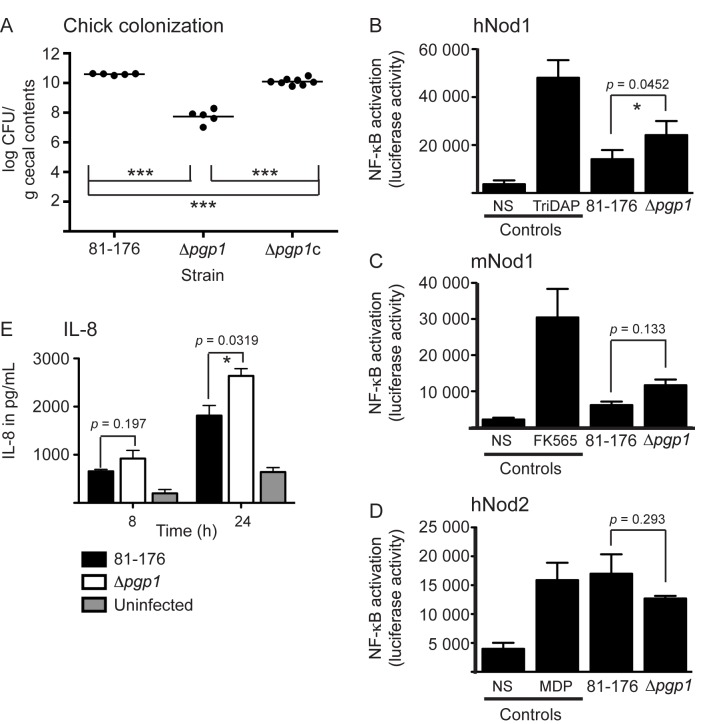


Corrected Body Text:

1. Legend to Figure 6:

The asterisk (*) indicates a statistically significant difference using the unpaired Student’s t-test, with * or *** indicating p < 0.05 or p < 0.001, respectively.

Replace with:

The asterisk (*) indicates a statistically significant difference using the unpaired (A, E) or paired (B-D) Student's t-test, with * or *** indicating p<0.05 or p<0.001, respectively.

2. Results:

We found that PG from the Δpgp1 mutant produced a statistically significant increase in mNod1 activation compared to wild type. In contrast, Δpgp1 mutant PG did not produce a statistically significant change in stimulation of hNod1, despite a modest increase from wild type.

Replace text with:

We found that PG from the Δpgp1 mutant produced a statistically significant increase in hNod1 activation compared to wild type. In contrast, Δpgp1 mutant PG did not produce a statistically significant change in stimulation of mNod1, despite a modest increase from wild type.

3. Discussion:

Using these assays, only mNod1 exhibited a statistically significant difference in activation by Δpgp1 PG compared to wild type.

Replace text with:

Using these assays, only hNod1 exhibited a statistically significant difference in activation by Δpgp1 PG compared to wild type, as predicted from the higher amount of tripeptides in the Δpgp1 PG. Despite a decrease in tetrapeptides and dipeptides, no significant changes in mNod1 and Nod2 were detected.

4. Discussion:

Thus while the increase in IL-8 secretion in response to Δpgp1 may be due to the modest increase in hNod1 activation observed in our luciferase assays, it cannot be ruled out that deletion of pgp1 leads to a change in another, as-yet unidentified factor stimulating IL-8 expression. Future work is planned to address these hypotheses.

Replace text with:

Thus while the increase in IL-8 secretion in response to Δpgp1 may be due to the increase in hNod1 activation observed in our luciferase assays, it cannot be ruled out that deletion of pgp1 leads to a change in another, as-yet unidentified factor stimulating IL-8 expression. Future work is planned to address these hypotheses.

(deleted the word modest)

