# Supplementary figures and images for: Peptidoglycan-Modifying Enzyme Pgp1 Is Required for Helical Cell Shape and Pathogenicity Traits in Campylobacter jejuni
Source: PLoS Pathog. 2012 Mar 22;8(3):e1002602. doi: 10.1371/journal.ppat.1002602 (PMC3310789; doi:10.1371/journal.ppat.1002602)

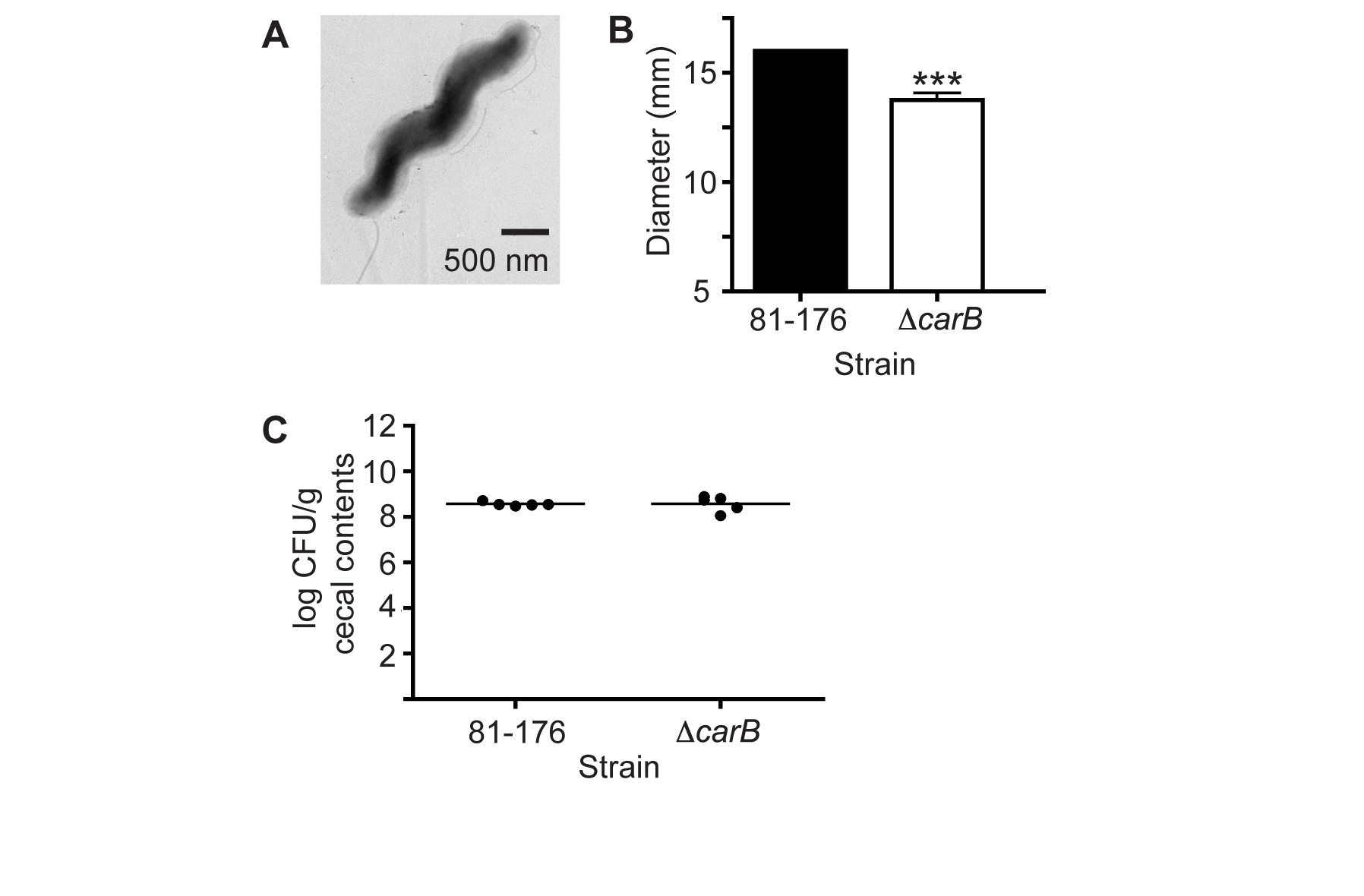

Supplement: Figure S1 — C. jejuni Δ carB has a motility defect, but is unaffected for chick colonization. A, negatively stained TEM images of ΔcarB showing the full-length flagella and helical morphology. B, ΔcarB exhibits a slight motility defect, as assayed by measuring halo diameters in soft agar plates. Standard error of the mean was calculated from 8 measurements. The asterisks (***) indicate a statistically significant difference (p<0.001) using the unpaired Student's t-test. C, ΔcarB shows no defect for chick colonization. Each point represents the log CFU/g cecal contents of an individual chick 6 days following infection with 104 CFUs of the indicated C. jejuni strains. The geometric mean is denoted by a black bar. (TIF) [file ppat.1002602.s001.tif]

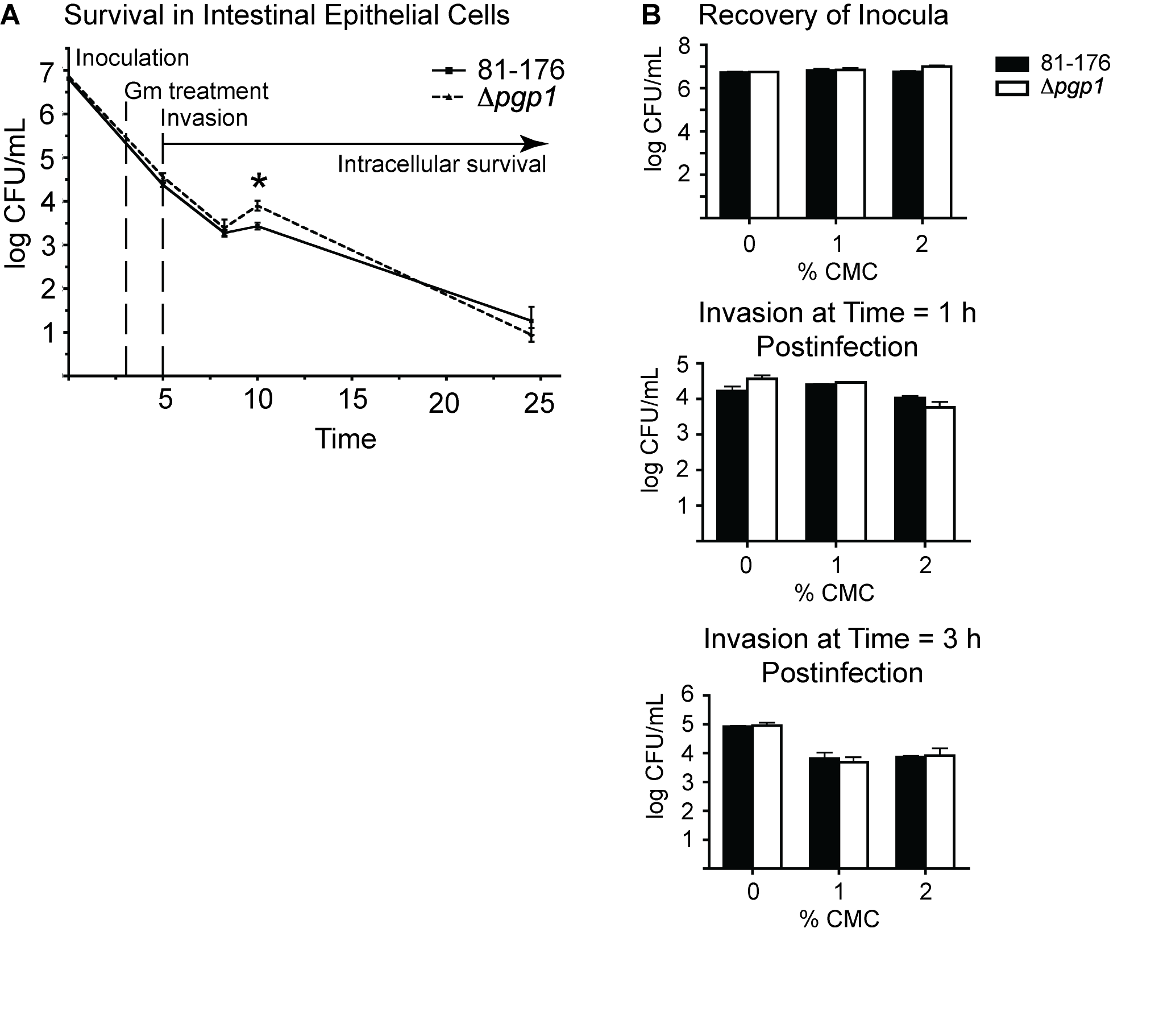

Supplement: Figure S2 — C. jejuni Δ pgp1 shows slightly enhanced intracellular survival, but no defect in invasion in the presence of carboxymethylcellulose (CMC). A, a gentamicin (Gm) protection assay was used to assess the invasion and intracellular survival ability of Δpgp1 in Caco-2 intestinal epithelial cells. Gm was added 3 h post-infection with bacterial strains. After 2 h the Gm was washed off and the cells were incubated with fresh MEM containing 3% FBS and a low dose of Gm. B, infections were carried out in the presence of MEM containing 1% and 2% CMC to examine the ability of Δpgp1 to invade INT407 epithelial cells in higher viscosity media. CFUs were determined for each well by lysing the cells with water and plating the dilutions onto MH-TV plates. Standard errors of the mean were calculated from triplicate readings and are representative of three independent experiments. The asterisk (*) indicates a statistically significant difference (p<0.05) using the unpaired Student's t-test. (TIF) [file ppat.1002602.s002.tif]
