# Supplementary material for: Peptidoglycan-Modifying Enzyme Pgp1 Is Required for Helical Cell Shape and Pathogenicity Traits in Campylobacter jejuni
Source: PLoS Pathog. 2012 Mar 22;8(3):e1002602. doi: 10.1371/journal.ppat.1002602 (PMC3310789; doi:10.1371/journal.ppat.1002602)
Supplement: Table S1 — BLAST results for the C. jejuni 81-176 1344/pgp1 gene product. (DOC) [file ppat.1002602.s003.doc]

**Table S1.** Summary of BLAST results with an E-value ≤ 1e-5 for the *C. jejuni* 81-176 *1344/pgp1* gene product.

| Species | Class | No. of BLAST hits | Gene Designation | Lowest E-value | % Identity/  % Similarity | Shape | Predicted signal peptide using SignalP |
| --- | --- | --- | --- | --- | --- | --- | --- |
| *Campylobacter jejuni* subsp. *jejuni* 81-176 | ε-Proteobacteria | 1 | YP_001001002.1 | 0 | 100/100 | Helical | Yes |
| *Campylobacter jejuni* subsp. *jejuni* HB93-13 | ε-Proteobacteria | 1 | ZP_01070980.1 | 0 | 99/99 | Helical | Yes |
| *Campylobacter jejuni* subsp. *jejuni* 81116 | ε-Proteobacteria | 1 | YP_001482837.1 | 0 | 98/99 | Helical | Yes |
| *Campylobacter jejuni* subsp. *jejuni* 260.94 | ε-Proteobacteria | 1 | ZP_01069014.1 | 0 | 98/99 | Helical | Yes |
| *Campylobacter jejuni* subsp. *jejuni* NTC11168 | ε-Proteobacteria | 1 | YP_002344733.1 | 0 | 98/98 | Helical | Yes |
| *Campylobacter jejuni* RM1221 | ε-Proteobacteria | 1 | YP_179519.1 | 0 | 98/99 | Helical | Yes |
| *Campylobacter jejuni* subsp. *jejuni* CG8421 | ε-Proteobacteria | 1 | ZP_03223563.1 | 0 | 98/99 | Helical | Yes |
| *Campylobacter jejuni* subsp. *jejuni* 1336 | ε-Proteobacteria | 1 | ZP_06374205.1 | 0 | 97/98 | Helical | Yes |
| *Campylobacter jejuni* subsp. *dolyei* 269.97 | ε-Proteobacteria | 1 | YP_001397574.1 | 0 | 96/98 | Helical | Yes |
| *Campylobacter jejuni* subsp. *jejuni* 414 | ε-Proteobacteria | 1 | ZP_06372473.1 | 0 | 95/96 | Helical | Yes |
| *Campylobacter coli* RM2228 | ε-Proteobacteria | 1 | ZP_00367395.1 | 0 | 92/95 | Helical | Yes |
| *Campylobacter coli* JV20 | ε-Proteobacteria | 1 | ZP_07400774.1 | 0 | 91/95 | Helical | Yes |
| *Campylobacter jejuni* subsp. *jejuni* CG8486 | ε-Proteobacteria | 1 | ZP_01810378.1 | 0 | 98/99 | Helical | Yes |
| *Campylobacter jejuni* subsp. *jejuni* CF93-6 | ε-Proteobacteria | 2 | ZP_01068269.1  ZP_01068323.1 | 0  4e-24 | 98/99  96/96 | Helical | Yes  No |
| *Campylobacter jejuni* subsp. *jejuni* 84-25 | ε-Proteobacteria | 2 | ZP_01099371.1  ZP_01099270.1 | 0  1e-30 | 98/99  100/100 | Helical | Yes  No |
| *Campylobacter upsaliensis* RM3195 | ε-Proteobacteria | 1 | ZP_00370146.1 | 0 | 77/87 | Helical | Yes |
| *Campylobacter lari* RM2100 | ε-Proteobacteria | 1 | YP_002576089.1 | 3e-127 | 66/78 | Helical | Yes |
| *Campylobacter fetus* subsp. *fetus* 82-40 | ε-Proteobacteria | 1 | YP_891418.1 | 2e-122 | 50/66 | Helical | No |
| *Campylobacter curvus* 525.92 | ε-Proteobacteria | 1 | YP_001408994.1 | 6e-111 | 45/62 | Helical, curved rods | Yes |
| *Campylobacter concisus* 13826 | ε-Proteobacteria | 1 | YP_001467262.1 | 1e-108 | 51/70 | Helical | Yes |
| *Desulfovibrio desulfuricans* subsp. *desulfuricans* str. G20 | δ-Proteobacteria | 1 | YP_386559.1 | 3e-99 | 46/64 | Vibrioid | Yes |
| *Arcobacter nitrofigilis* DSM 7299 | ε-Proteobacteria | 1 | YP_003657196.1 | 5e-99 | 45/65 | Helical | Yes |
| Campylobacterales bacterium GD 1 | ε-Proteobacteria | 2 | ZP_05072078.1  ZP_05072489.1 | 1e-98  1e-58 | 47/65  35/56 |  | No |
| *Campylobacter fetus* subsp. *venerealis* str. Azul-94 | ε-Proteobacteria | 1 | ZP_06009950.1 | 4e-97 | 57/73 | Helical | Yes |
| *Desulfovibrio vulgaris* str. 'Miyazaki F' | δ-Proteobacteria | 1 | YP_002436677.1 | 2e-96 | 42/62 | Vibrioid | Yes |
| *Desulfovibrio vulgaris* str. Hildenborough | δ-Proteobacteria | 1 | YP_012405.1 | 6e-96 | 45/63 | Vibrioid | Yes |
| *Desulfovibrio vulgaris* DP4 | δ-Proteobacteria | 1 | YP_965643.1 | 3e-95 | 45/63 | Vibrioid | Yes |
| *Desulfovibrio magneticus* RS-1 | δ-Proteobacteria | 1 | YP_002952508.1 | 5e-93 | 40/60 | Vibrioid | Yes |
| *Desulfovibrio desulfuricans* subsp. *desulfuricans* str. ATCC 27774 | δ-Proteobacteria | 1 | YP_002480452.1 | 2e-92 | 43/63 | Vibrioid | Yes |
| *Desulfovibrio* sp. 3_1_syn3 | δ-Proteobacteria | 1 | ZP_07356065 | 2e-92 | 42/57 | Vibrioid | No |
| *Desulfovibrio* sp. FW1012B | δ-Proteobacteria | 1 | ZP_06371045.1 | 2e-92 | 41/60 | Vibrioid | Yes |
| *Desulfovibrio fructosovorans* JJ | δ-Proteobacteria | 1 | ZP_07333166.1 | 1e-91 | 39/61 | Vibrioid | Yes |
| *Helicobacter pylori* 35A | ε-Proteobacteria | 1 | ZP_07019319.1 | 7e-90 | 41/60 | Helical | Yes |
| *Helicobacter pylori* 98-10 | ε-Proteobacteria | 1 | ZP_03438765.1 | 6e-89 | 41/60 | Helical | Yes |
| *Helicobacter pylori* Shi470 | ε-Proteobacteria | 1 | YP_001909868.1 | 5e-89 | 41/60 | Helical | Yes |
| *Helicobacter pylori* HPKX_438_AG0C1 | ε-Proteobacteria | 3 | ZP_03240194.1  ZP_03245558.1  ZP_03243971.1 | 6e-89  8e-12  7e-9 | 41/60  64/77  39/61 | Helical | Yes  No  No |
| *Helicobacter mustelae* 12198 | ε-Proteobacteria | 2 | YP_003516363.1 | 1e-88 | 44/61 | Helical | No |
| *Helicobacter pylori* G27 | ε-Proteobacteria | 2 | YP_002265985.1 | 2e-88 | 41/61 | Helical | Yes |
| *Helicobacter pylori* 26695 | ε-Proteobacteria | 2 | NP_207866.1 | 3e-88 | 40/60 | Helical | Yes |
| *Helicobacter pylori* J99 | ε-Proteobacteria | 2 | NP_223069.1 | 3e-88 | 41/60 | Helical | Yes |
| *Helicobacter acinonychis* str. Sheeba | ε-Proteobacteria | 2 | YP_664945.1 | 2e-87 | 42/60 | Helical | No |
| *Helicobacter pylori* P12 | ε-Proteobacteria | 2 | YP_002301006.1 | 2e-87 | 40/59 | Helical | Yes |
| *Wolinella succinogenes* DSM 1740 | ε-Proteobacteria | 1 | NP_906487.1 | 7e-87 | 42/60 | Helical, curved or straight | Yes |
| *Helicobacter pylori* HPAG1 | ε-Proteobacteria | 1 | YP_627113.1 | 1e-86 | 40/60 | Helical | Yes |
| *Helicobacter pylori* B38 | ε-Proteobacteria | 1 | YP_003057169.1 | 1e-86 | 40/59 | Helical | No |
| *Helicobacter pylori* B8 | ε-Proteobacteria | 1 | YP_003729215.1 | 6e-86 | 40/59 | Helical | Yes |
| *Helicobacter hepaticus* ATCC 51449 | ε-Proteobacteria | 1 | NP_860063.1 | 4e-81 | 39/56 | Helical | Yes |
| *Sulfurospirillum deleyianum* DSM 6946 | ε-Proteobacteria | 1 | YP_003304742.1 | 4e-76 | 38/59 | Helical | Yes |
| *Wolinella succinogenes* DSM1740 | ε-Proteobacteria | 1 | NP_906997.1 | 6e-75 | 38/59 | Helical, curved or straight | Yes |
| *Helicobacter cinaedi* CCUG 18818 | ε-Proteobacteria | 1 | ZP_03659062.1 | 5e-73 | 38/56 | Helical | Yes |
| *Sulfurimonas denitrificans* DSM 1251 | ε-Proteobacteria | 1 | YP_393892.1 | 6e-72 | 40/60 | Slightly curved or helical | Yes |
| *Nautilia profundicola* AmH | ε-Proteobacteria | 1 | YP_002607827.1 | 8e-72 | 37/62 | Slightly curved rods | Yes |
| *Arcobacter butzleri* RM4018 | ε-Proteobacteria | 1 | YP_001489842.1 | 1e-68 | 39/58 | Helical | Yes |
| *Helicobacter canadensis* MIT 98-5491 | ε-Proteobacteria | 1 | ZP_03655839.1 | 6e-66 | 36/56 | Curved rod | Yes |
| *Helicobacter winghamensis* ATCC BAA-430 | ε-Proteobacteria | 1 | ZP_04583508.1 | 9e-61 | 35/55 | Helical | Yes |
| *Helicobacter pullorum* MIT 98-5489 | ε-Proteobacteria | 1 | ZP_04808275.1 | 3e-60 | 36/54 | Curved rod | Yes |
| *Helicobacter pylori* B128 | ε-Proteobacteria | 2 | ZP_03437104.1  ZP_03437105.1 | 2e-47  5e-30 | 44/62  37/55 | Helical | Yes  No |
| Bacterium S5 | Unclassified bacterium | 1 | ZP_06403445.1 | 6e-35 | 30/46 |  | Yes |
| Denitrovibrio acetiphilus DSM 12809 | Deferribacteres (class) | 1 | YP_003505695.1 | 4e-34 | 40/57 | Vibriod | Yes |
| Deferribacter desulfuricans SSM1 | Deferribacteres (class) | 1 | YP_003496203.1 | 5e-34 | 40/59 | Curved rod or helical | Yes |
| Hydrogenivirga sp. 128-5-R1-1 | Aquificae | 1 | ZP_02180097.1 | 1e-31 | 39/53 | Straight, curved and filamentous rods | No |
| Persephonella marina EX-H1 | Aquificae | 1 | YP_002731141.1 | 2e-28 | 32/50 | Straight rod (appears curved in ) | Yes |
| Desulfarculus baarsii DSM 2075 | δ-Proteobacteria | 1 | YP_003807819.1 | 5e-28 | 36/55 | Vibrioid | Yes |
| Caminibacter mediatlanticus TB-2 | ε-Proteobacteria | 2 | ZP_01872282.1  ZP_01871401.1 | 8e-28  4e-20 | 40/58  34/55 | Rod | Yes  No |
| Nitratiruptor sp. SB155-2 | ε-Proteobacteria | 1 | YP_001356034.1 | 2e-26 | 37/52 | Rod | Yes |
| Desulfococcus oleovorans Hxd3 | δ-Proteobacteria | 1 | YP_001530083.1 | 2e-25 | 33/51 | Rods, irregular shaped cocci | Yes |
| Desulfovibrio salexigens DSM 2638 | δ-Proteobacteria | 1 | YP_002992815.1 | 6e-23 | 35/53 | Vibrioid | Yes |
| Desulfovibrio aespoeensis Aspo-2 | δ-Proteobacteria | 1 | ZP_06232544.1 | 4e-20 | 33/51 | Vibrioid | Yes |
| Haloterrigena turkmenica DSM 5511 | Halobacteria (Euryarchaeotes) | 3 | YP_003401886.1  YP_003406410.1  YP_003402697.1 | 2e-5  2e-5  6e-5 | 30/55  28/55  28/54 | Ovoid, coccoid and rod shaped | Yes  Yes  Yes |
