# Supplementary material for: Peptidoglycan-Modifying Enzyme Pgp1 Is Required for Helical Cell Shape and Pathogenicity Traits in Campylobacter jejuni
Source: PLoS Pathog. 2012 Mar 22;8(3):e1002602. doi: 10.1371/journal.ppat.1002602 (PMC3310789; doi:10.1371/journal.ppat.1002602)
Supplement: Table S2 — Summary of Δ pgp1 phenotypes tested that were similar to wild type. (DOC) [file ppat.1002602.s004.doc]

**Table S2.** Summary of ∆*pgp1* phenotypes tested that were similar to wild type.

|  | **Phenotype** | **Experiment** |
| --- | --- | --- |
| **Growth-related** | Growth curve | Growth over time measured by OD and CFU/mL |
|  | Anaerobic survival | Anaerobic atmosphere broth survival |
|  | Survival at low and high pH | Growth on plates and in broth at pH 4.0, 5.0, 6.0, 7.0, 8.0 and 9.0 |
|  | Heat shock | Growth in broth at 45 °C |
|  | Autoagglutination | Measurement of the OD of standing cultures at room temperature over 24 h |
|  | Osmotolerance | Growth on plates containing 0.8% NaCl |
| **Cell surface carbohyrates &** | LOS | Silver stained SDS-PAGE gels of Hitchcock & Brown LOS preparations |
| **proteins** | Capsule | Alcian blue stained SDS-PAGE and Penner immunoblotting of Hitchcock & Brown LOS preparations |
|  | Membrane protein profile | SDS-PAGE of inner and outer membrane proteins (as well as cytoplasmic and periplasmic proteins) |
|  | Cell surface hydrophobicity | Hexadecane partitioning and ammonium sulfate precipitation |
| **Pathogenesis-related** | Serum sensitivity | Survival in 10% human serum |
|  | Adherence, invasion and intracellular survival in macrophages cell lines | *In vitro* gentamicin protection assay with Raw264.7 and Thp-1 cell lines |
| **Antimicrobial resistance** | Tris | MIC |
|  | EDTA | MIC |
|  | Divalent cations | MIC: MgCl_2_, KCl, CaCl_2_ |
|  | Porcine bile salts | Growth on plates containing 2.5% and 5% bile |
|  | Deoxycholate | MIC |
|  | Lysozyme, lysozyme + EDTA | MIC |
|  | Detergents | MIC: SDS, Tween 20, Triton X-100 |
|  | Antimicrobial peptides | MIC: polymyxin B and protamine |
|  | Antibiotics | MIC: ampicillin, gentamicin, rifampicin |
