# Supplementary material for: Peptidoglycan-Modifying Enzyme Pgp1 Is Required for Helical Cell Shape and Pathogenicity Traits in Campylobacter jejuni
Source: PLoS Pathog. 2012 Mar 22;8(3):e1002602. doi: 10.1371/journal.ppat.1002602 (PMC3310789; doi:10.1371/journal.ppat.1002602)
Supplement: Table S3 — Molecular mass of C. jejuni 81-176 muropeptides (reduced form) separated by HPLC and analyzed by LTQ-FT-MS. (DOC) [file ppat.1002602.s005.doc]

**Table S3.** Molecular mass of *C. jejuni* 81-176 muropeptides (reduced form) separated by HPLC and analyzed by LTQ-FT-MS.

| Peak number^1^ | Muropeptide^2^ | Theoretical neutral mass (Da) | Neutral mass (Da)^3^ |
| --- | --- | --- | --- |
| 1 | Tri | 870.3706 | 870.3876 |
| 2 | Tetra | 941.4077 | 941.4204 |
| 3 | Di | 698.2858 | 698.2781 |
| 4 | Tri-Ac | 912.3812 | 912.3919 |
| 5 | Tetra-Ac | 983.4183 | 983.4362 |
| 6 | Di-Ac | 740.2964 | 740.3117 |
| 7 | TetraTri | 1793.7677 | 1793.8086 |
| 8 | TetraPentaGly^5^ | 1921.8263 | 1921.8820 |
| 9 | TetraTetra | 1864.8049 | 1864.8248 |
| 10 | TetraTri-Ac | 1835.7783 | 1835.8296 |
| 11 | TetraTetraTri | 2717.1649 | 2717.2332 |
| 12 | Tetra Tetra-Ac | 1906.8154 | 1906.8400 |
| 13 | TetraTetraTetra | 2788.2020 | 2788.3112 |
| 14 | TetraTriAnh I | 1773.7415 | 1773.7680 |
| 15 | TetraTriAnh II | 1773.7415 | 1773.7800 |
| 16 | TetraTetraAnh I | 1844.7786 | 1844.8440 |
| 17 | TetraTetraAnh II | 1844.7786 | 1844.8390 |
| 18 | TetraTetraTetraAnh I | 2768.1758 | 2768.1758 |

^1^ Peak numbers correspond to peaks labeled in the HPLC chromatogram shown in Fig. 4A.

^2^Muropeptides are named according to Glauner *et al*. (1998) and are depicted in Fig. 4E. Di, disaccharide dipeptide (disaccharide = β 1,4-linked N-acetylglucosamine-N-acetylmuramic acid); Tri, disaccharide tripeptide; Tetra, disaccharide tetrapeptide; Penta, disaccharide pentapeptide; Gly, glycine in position 5 of a peptide side chain; Ac, O-acetyl groups at the C-6 hydroxyl group of MurNAc; Anh, 1,6-anhydromuramic acid. Disaccharides are linked to form dimers or trimers by D,D- crosslinks between amino acids 4 (D-Ala) and 3 (*meso-*DAP).

^2^ Neutral masses were calculated from the masses of mono- or multiple charged protonated compounds (H^+^-form). Most compounds were also present in the Na^+^-and K^+^-form.
