# Supplementary material for: Peptidoglycan-Modifying Enzyme Pgp1 Is Required for Helical Cell Shape and Pathogenicity Traits in Campylobacter jejuni
Source: PLoS Pathog. 2012 Mar 22;8(3):e1002602. doi: 10.1371/journal.ppat.1002602 (PMC3310789; doi:10.1371/journal.ppat.1002602)
Supplement: Table S4 — Muropeptide compositional analysis used to determine the values in Table 1 . (DOC) [file ppat.1002602.s006.doc]

**Table S4.** Muropeptide composition of *C. jejuni* wild-type 81-176, the ∆*pgp1* mutant, ∆*pgp1* complement (∆*pgp1c*), and *pgp1* overexpression (81-176+*pgp1*) strains, and the resultant ∆*pgp1* PG profiles of Pgp1 activity assays consisting of ∆*pgp1* PG incubated without enzyme, with Pgp1 in the presence of ZnCl_2_, and with Pgp1 without ZnCl_2_ but with EDTA.

|  |  | **% Peak area** | | | | | | | |
| --- | --- | --- | --- | --- | --- | --- | --- | --- | --- |
|  |  | **In *C. jejuni* strains** | | | |  | **Following incubation of ∆*pgp1* PG** | | |
| **Peak number** | **Muropeptide** | **81-176** | **∆*pgp1*** | **∆*pgp1*c** | **81-176 + *pgp1*** |  | **∆*pgp1* PG**  **- Pgp1** | **∆*pgp1* PG**  **+ Pgp1+ ZnCl_2_** | **∆*pgp1* PG**  **+ Pgp1 + EDTA** |
| 1 | Tri | 9.3 | 27.1 | 2.8 | 1.9 |  | 29.6 | 1.5 | 29.4 |
| 2 | Tetra | 16.4 | 2.7 | 17.3 | 18.6 |  | 2.3 | 4.4 | 5.1 |
| 3 | Di | 11.9 | 4.2 | 16.2 | 15.4 |  | 5.0 | 27.3 | 4.8 |
| 4 | Tri-Ac | 0.5 | 3.5 | 0.3 | 0.3 |  | 0.2 | 0.3 | 1.2 |
| 5 | Tetra-Ac | 0.9 | 0.0 | 0.6 | 1.6 |  | 0.3 | 0.3 | 0.2 |
| 6 | Di-Ac | 1.7 | 1.0 | 3.0 | 1.9 |  | 0.1 | 1.7 | 0.5 |
| 7 | TetraTri | 9.3 | 20.9 | 4.3 | 2.4 |  | 26.1 | 25.0 | 23.1 |
| 8 | TetraPentaGly5 | 1.4 | 0.7 | 1.5 | 1.8 |  | 0.8 | 0.8 | 0.7 |
| 9 | TetraTetra | 21.2 | 8.7 | 26.0 | 27.8 |  | 9.3 | 8.6 | 7.7 |
|  | TetraAnh |  |  |  |  |  | 0.2 | 5.7 | 6.1 |
| 10 | TetraTri-Ac | 1.7 | 4.7 | 0.6 | 0.3 |  | 0.1 | 1.9 | 1.5 |
| 11 | TetraTetraTri | 0.8 | 1.7 | 0.6 | 0.3 |  | 2.7 | 2.4 | 2.1 |
| 12 | TetraTetra-Ac | 2.8 | 1.4 | 3.0 | 4.9 |  | 0 | 0.7 | 0 |
| 13 | TetraTetraTetra | 1.8 | 0.6 | 2.2 | 2.4 |  | 1.4 | 1.3 | 1.6 |
| 14 | TetraTriAnh I | 1.1 | 2.4 | 0.6 | 0.4 |  | 2.3 | 2.6 | 2.4 |
| 15 | TetraTriAnh II | 1.7 | 5.0 | 0.9 | 0.3 |  | 6.2 | 3.3 | 1.8 |
| 16 | TetraTetraAnh I | 3.8 | 1.5 | 4.5 | 4.9 |  | 1.2 | 1.1 | 0.9 |
| 17 | TetraTetraAnh II | 3.6 | 1.5 | 4.5 | 4.3 |  | 1.9 | 0 | 0 |
| 18 | TetraTetraTetraAnh | 3.9 | 1.5 | 4.3 | 4.8 |  | 1.9 | 1.4 | 1.1 |
| 1 - 18 | All known | 93.6 | 88.9 | 93.0 | 94.1 |  | 91.6 | 89.7 | 90.2 |
