# Supplementary material for: Peptidoglycan-Modifying Enzyme Pgp1 Is Required for Helical Cell Shape and Pathogenicity Traits in Campylobacter jejuni
Source: PLoS Pathog. 2012 Mar 22;8(3):e1002602. doi: 10.1371/journal.ppat.1002602 (PMC3310789; doi:10.1371/journal.ppat.1002602)
Supplement: Table S5 — PG muropeptide composition of C. jejuni wild-type strain 81-176 in comparison to H. pylori and E. coli . (DOC) [file ppat.1002602.s007.doc]

**Table S5.** Summary of PG muropeptide composition of *C. jejuni* wild-type strain 81-176 in comparison to *H. pylori* strain G27 and the published muropeptide map of exponentially grown *E. coli* KN126 . Numbers represent the percent area of each muropeptide calculated to give a total of 100%.

| **Muropeptide species^1^** | ***Campylobacter jejuni* 81-176** | ***Helicobacter pylori* G27** | ***Escherichia coli* KN126 (exponential growth phase)**^2^ |
| --- | --- | --- | --- |
| **Monomers (Total)** | **43.3** | **58.1** | **54.5** |
| Di | 14.5 | 2.6 | 2.2 |
| Tri | 10.5 | 4.21 | 12.8 |
| Tetra | 18.4 | 9.8 | 37.4 |
| Tetra (Gly^4^) | -^3^ | - | 1.7 |
| Penta | - | 37.0 | 0.07 |
| Penta (Gly^5^) | - | 4.5 | 0.3 |
|  |  |  |  |
| **Dimers (Total)** | **49.7** | **41.9** | **41.2** |
| Tri Tri | - | ­- | 0.7 |
| Tetra Tri | 14.7 | 4.5 | 9.1 |
| Tetra Tri (Gly^4^) | - | - | 0.07 |
| Tetra Tetra | 33.5 | 15.7 | 29.4 |
| Tetra Tetra (Gly^4^) | - | - | 1.5 |
| Tetra Penta | - | 19.5 | 0.2 |
| Tetra Penta (Gly^5^) | 1.5 | 2.1 | 0.3 |
|  |  |  |  |
| **Trimers (total)** | **6.9** | **-** | **4.2** |
| Tetra Tetra Tri | 0.8 | - | 1.2 |
| Tetra Tetra Tetra | 6.1 | - | 3.0 |
|  |  |  |  |
| **Tetramers (Total)** | **n/a** | **-** | **0.1** |
|  |  |  |  |
| Dipeptides (Total) | 14.5 | 2.6 | 2.2 |
| Tripeptides (Total) | 18.1 | 6.5 | 18.5 |
| Tetrapeptides (Total) | 66.7 | 38.7 | 78.6 |
| Pentapeptides (Total) | 0.7 | 52.2 | 0.6 |
| Anhydro chain ends (Total) | 6.8 | 10.0 | 4.8 |
| Lys-Arg(Total)^4^ | - | - | 8.9 |
|  |  |  |  |
| Average chain length | 14.7 | 10.0 | 25.8 |
|  |  |  |  |
| Degree of cross-linkage | 29.5 | 21.0 | 25.1 |
| D,D-crosslinks | 29.5 | 21.0 | 22.4 |
| L,D-crosslinks | - | - | 2.8 |
