# Supplementary material for: Peptidoglycan-Modifying Enzyme Pgp1 Is Required for Helical Cell Shape and Pathogenicity Traits in Campylobacter jejuni
Source: PLoS Pathog. 2012 Mar 22;8(3):e1002602. doi: 10.1371/journal.ppat.1002602 (PMC3310789; doi:10.1371/journal.ppat.1002602)
Supplement: Text S1 — Supplemental materials and methods. (DOC) [file ppat.1002602.s008.doc]

**Text S1. SUPPLEMENTAL MATERIALS AND METHODS**

**Random *in vitro* transposon mutagenesis of *C jejuni* using the *mariner* transposon**

*Purification of MBP-Himar1*

The MBP-Himar1 transposase was purified according to a modified protocol from Akerley and Lampe (2002) and instructions from the manufacturer (pMAL Protein Fusion and Purification System Instruction Manual; NEB). An overnight culture of *E. coli* TB1 containing the plasmid pMALC9 grown in LB and Ap 100 µg/ml at 37 °C was subcultured 1/50 into 100 mL fresh LB containing Ap and 0.2% (w/v) glucose and growth at 37 °C was continued. Glucose is required to repress amylase expression in *E. coli* that may interfere with binding to the amylose column. At an approximate OD of 0.5, protein expression was induced with 0.3 mM IPTG and incubation was continued for 2 h. The bacterial cells were harvested by centrifugation and frozen until needed. The cell pellet was resuspended in column buffer (CB; mM Tris-HCl (pH 7.4), 200 mM NaCl, 1 mM EDTA) and sonicated for 2 min (10 sec pulse ON / 10 sec pulse OFF). The cellular debris was removed by centrifugation for 10 min at 13 000 rpm, and protease inhibitors (Roche complete, mini EDTA-free protease inhibitor cocktail tablets) were added to the supernatant.

The amylose resin (NEB) was prepared by washing with transposase wash buffer (TWB; 20 mM Tris-HCl (pH 7.4), 200 mM NaCl, 1 mM EDTA, 2 mM DTT, 10 % glycerol), as described by the manufacturer. The lysate was diluted to a final volume of 5 mL using CB. The amylase resin was added to the lysate and incubated shaking overnight at 4 °C. The resin and lysate were added to an empty 5 mL column (Qiagen), and the flow-through collected by gravity flow. The column was washed 4 times with 2 mL TWB. A total of 0.4 mL of transposase elution buffer (TEB; 20 mM Tris-HCl (pH 7.4), 200 mM NaCl, 1 mM EDTA, 2 mM DTT, 10 % glycerol, 10 mM maltose) was added to the column, incubated for 5 min, and the elution fraction containing the purified transposase was collected. The transposase was aliquoted in 10 µl volumes and frozen at – 80 °C. The protein concentration was determined by Bradford assay (Biorad) (previous concentration was 0.16 mg/mL) and purity was assessed by SDS-PAGE.

*In vitro transposon* (Tn) *mutagenesis*

*In vitro* transposition reactions were performed as described . The transposition buffer (total volume of 80 µL) contained 10 % glycerol, 25 mM Hepes pH 8.0, 250 ug/mL BSA, 1 mM DTT, 100 mM NaCl, and 5 mM MgCl_2_ to which 2 µg *C. jejuni* 81-176 genomic DNA 100/G, 1 µg pFalcon or pEnterprise, and 0.5 µg transposase was added. The transposase was added last to initiate the reaction. The transposition reaction was incubated at 28°C for 4 h. The DNA was purified from the reaction mixture using Qiagen DNeasy columns according to the manufacturer’s instructions. The gaps at the Tn-chromosomal DNA junctions were repaired by treatment with DNA polymerase I, large (Klenow; NEB) and then T4 DNA ligase (NEB). The DNA from the ligation reaction was dialyzed on a 0.025 micron hydrophobic filter floating on dH_2_O for 20 min and then transformed by natural transformation into *C. jejuni* 81-176. Km-resistant (*solo* Tn) and Cm-resistant (*picard* Tn) clones were selected on MH-TV-Km and MH-TV-Cm plates, respectively. Approximately 3 000 – 4 000 single colonies from each round of mutagenesis were directly harvested from the plates and stored at –80°C. A total of 4 rounds of mutagenesis were carried out to construct the library. To confirm random Tn insertion, 10 of the colonies from each of the *solo* and *picard* Tn libraries were screened by Southern blot (data not shown).

*Tn mapping by random PCR*

The region flanking Tn insertion sites was amplified from genomic DNA of the Tn mutants using the CEKG primers and PCR protocol of Salama *et al.* (2004) . In a first PCR reaction, random primers CEKG2A, CEKG2B, or CEKG2C were used with either the mariner-2 or mariner-3 Tn specific primers to amplify Tn flanking sites. A second PCR was performed on a fivefold dilution of the first PCR reaction using the primers CEKG4 and mariner-IR-1. The reaction products were purified and sequenced using the MarOut3 primer. Fine mapping to determine the precise location of the Tn insertion was done with PCR primers designed to the specific gene into which the Tn had inserted and by sequencing the product.

**Construction of *C. jejuni* 81-176 Δ*pgp1* & Δ*1343* targeted deletion strains, and *pgp1* overexpression and complementation strains.**

The *pgp1* gene was PCR amplified with iProof (Biorad) from 81-176 genomic DNA using primers 1344Forw and 1344Rev. A polyA tag was added to the PCR product and it was ligated to a commercially available pGEM-T vector (Promega). The resulting construct pGEMT-*pgp1* was verified by restriction enzyme digestion and sequencing. Inverse PCR was performed on the resulting plasmid with primers 1344-1 and 1344-2 deleting 880 bp of the gene and ligated to the non-polar kanamycin resistance cassette (*aphA-3*) digested out of pUC18K-2 with *SmaI* to form pGEMT-*pgp1*Km. Orientation of the Km resistance cassette was verified by restriction enzyme digestion. *C. jejuni* 81-176 was naturally transformed with pGEMT-*pgp1*Km. 81-176 Δ*pgp1* mutant strains were selected by a Km^R^ phenotype and verified by PCR and restriction enzyme digestion.

To delete *1343*, the *1343* gene was PCR amplified from 81-176 genomic DNA with primers 1343-3 and 1343-4, polyA tagged and ligated to pGEM-T. The construct was verified by restriction enzyme digestion and sequencing. The *aphA3* cassette digested out of pUC18K-2 with *XbaI* and *KpnI* and ligated to the *XbaI* and *KpnI* digested inverse PCR product of pGEMT-1343 amplified with primers 1343-5 (*KpnI*) and 1343-6 (*XbaI*), deleting 775 bp of the gene. The pGEMT-1343Km plasmid was transformed by natural transformation into *C. jejuni* 81-176 and Δ*1343* mutant strains were selected by Km^R^ and verified by PCR and restriction enzyme digestion.

For overexpression and complementation studies of *pgp1*, the gene was PCR amplified from 81-176 genomic DNA and cloned into pRRC and pRRK [J. Ketley, unpublished; based on pRRC integration vectors of Karlyshev and Wren (2005)] with varying amounts of the upstream region. To generate pEF15 and pEF20, *pgp1* was PCR amplified with 1344-5 (*NheI*) and 1344-7 and the PCR product was digested with *NheI* and ligated to pRRC (for pEF15) and pRRK (for pEF20). The vectors were first digested with *MfeI*, blunted by filling in with Klenow, and further digested with *XbaI*. Plasmid pEF35R used for complementation was constructed by amplifying the *pgp1* gene with primers 1348-1 (*SpeI*) and 1344-15 (*SpeI*) and ligating the *NheI*-digested product to pRRC digested with *XbaI* and dephosphorylated. The resulting construct was verified for orientation by PCR and sequenced. Both pEF15 and pEF20 were cloned with the *pgp1* gene in the same orientation as the promoter for the antibiotic resistance cassette, while pEF35R was made in the reverse orientation (designated with an R). Plasmids were inserted into 81-176 and Δ*pgp1* by natural transformation and transformants selected on the appropriate antibiotics. Single insertions into the rRNA spacer region were verified by PCR with primers ak233, ak234, ak235 and primers cat-2 or aphA3-2 for pRRC and pRRK constructs, respectively.

**Expression and purification of Trx-His_6_-Pgp1**

*Construction of C. jejuni 81-176 Pgp1 expression and purification construct.*

The *pgp1* gene was PCR amplified with iProof (Biorad) from 81-176 genomic DNA using primers 1344-18 (*NcoI*) and 1344-8 (*XhoI*) including amino acids 16-464 of the protein, without the predicted signal peptide. The PCR product was digested with *NcoI* and *XhoI* sites included in the primers and ligated to a similarily digested pET32a(+) vector (Novagen). The *pgp1* gene was cloned in frame with the Trx-tag thioredoxin protein to improve solubility and His tag encoded by the vector. The construct was verified by digestion and sequencing and then transformed into BL21 [λDE3].

*Purification of Pgp1*

The Pgp1 protein was overexpressed from pEF46 in *E. coli* BL21 [λDE3]. This strain was grown at 38°C for 18 h in LB supplemented with Amp. The culture was diluted 1:50 in 500 ml of fresh medium, and incubation was continued until the culture reached an optical density of 0.6 at 600 nm. Expression of pEF46 was induced by adding isopropyl-1-thio-ß-D-galactopyranoside to the culture at a final concentration of 0.5 mM and continuing incubation at 38°C for another 3 h. The cells were harvested and then the pellet was frozen until required. The cell pellet was resuspended in a total of 30 ml of breaking buffer [0.2 M Tris-Cl, pH 7.6 containing 0.2 M NaCl, 0.001 M Mg(CH_3_COO)_2_, and 5% (v/v) glycerol] and the cells were lysed by homogenization. Unbroken cells and cell debris were removed by two centrifugation steps (15 min at 15,000 x *g*). The lysate was combined with 2 ml of 50% Ni^2+^-nitrilotriacetic acid (NTA) suspension (QIAGEN), and the mixture was incubated for 2 h at 4°C on a rotary shaker. The lysate-Ni^2+^-NTA mixture was then loaded into a disposable plastic column (10 ml) with elution by gravity flow. The column was washed twice with 4 column volumes of wash buffer [0.05 M NaH_2_PO_4_, pH 8.0 containing 0.3 M NaCl and 35 mM imidazole]. The protein was eluted in four fractions of one column volume of elution buffer [0.05 M NaH_2_PO_4_, pH 8.0 containing 0.3 M NaCl, 250 mM imidazole, 20% (v/v) glycerol]. The fractions were examined by SDS-PAGE, and those with the highest amount of highest purity protein were pooled and dialyzed against 0.05 M Tris-Cl, pH 7.5 containing 0.01 M ZnCl_2_, 0.3 M NaCl and 20% glycerol. Typical extracts contained 5.5 mg/ml protein.

***In vitro* invasion and intracellular survival in epithelial and macrophage cell lines**

The human epithelial cell lines T84, CaCo2 and INT407 and the murine RAW 264.7 and human Thp-1 macrophage cell lines were used for *C. jejuni* infections. Media used for growth of the cell lines was as directed by the ATCC. Cells were seeded into 24-well tissue culture plates at semiconfluence (~5 x 10^5^ cells/ml for CaCo2 cells, ~1 x 10^5^ cells/ml for all other cell lines) and allowed to grow for 20-24 h prior to infection (except for the THP-1 cells). The RAW264.7 macrophage cell line was seeded in the presence of 1 ug/mL LPS (LPS from *Salmonella enterica* serotype typhimurium; Sigma) to prime the macrophages. THP-1 human monocytes were differentiated into macrophages with the addition of 100 nM phorbol 12-myristate 13-acetate (PMA; Sigma) for 2 days prior to the start of the experiment. Infections were carried out as described , except that *C. jejuni* strains from an 18h shaking broth culture were used for the inoculation, 250 µg/ml gentamicin was used for the gentamicin treatment, and after 5 h to assay intracellular survival in remaining wells, fresh medium was added containing 10 µg/ml gentamicin and 3% FBS for epithelial cells, and the cells were reincubated.

To test the levels of invasion in media of higher viscosity to mimic intestinal mucus, carboxymethylcellulose (CMC; Sigma) was used. Infections were carried out as above using the INT407 cell line. The bacterial inoculum was added in MEM containing 0, 0.6 (141 cP), 1, and 2% CMC. Levels of adhered and invaded bacteria at 1 and 3 h timepoints were determined by washing and lysing the cells, and plating for CFU/mL as described .

Bacterial strains or plasmids used in this study.

| **Strain or Plasmid** | **Genotype, serotype or description** | **Reference or Source** |
| --- | --- | --- |
| ***C. jejuni*** |  |  |
| 81-176 | Wild type isolated from a diarrheic patient |  |
| Δ*pgp1* | 81-176 *pgp1*::*aphA3*; Km^R^ | This study |
| Δ*1343* | 81-176 *1343*::*aphA3*; Km^R^ | This study |
| Δ*carB* | 81-176 *carB*::*aphA3*; Km^R^ |  |
| 81-176+*pgp1* | 81-176 *rrn::pgp1* (from pEF20) | This study |
| Δ*pgp1c* | 81-176 Δ*1344 rrn::pgp1* (from pEF35R) | This study |
| ***E. coli*** |  |  |
| DH5α | F^-^, φ80d *deoR lacZ*Δ*M15 endA1 recA1 hsdR17*(r_K_-m_K_+) *supE44 thi-1 gyrA96 relA1* Δ*(lacZYA-argF) U169* | Invitrogen |
| DH5α [λ *pir*] | F^-^, φ80d *deoR lacZ*Δ*M15 endA1 recA1 hsdR17*(r_K_-m_K_+) *supE44 thi-1 gyrA96 relA1* Δ*(lacZYA-argF) U169* [λ *pir*] |  |
| TB1 | F^-^, *ara* Δ *(lac-proAB) [*Φ*80dlac* Δ *(lacZ)M15] rpsL*(Str^R^) *thi hsdR* | NEB |
| BL21[λ DE3] | F^-^ omp*T* hsd*S_B_*(r_B_^–^, m_B_^–^) gal dcm [λ DE3] | Novagen |
| **Plasmids** |  |  |
| pMALC9 | pMAL-cri expressing the *Himar1* transposase cloned as a maltose binding protein (MBP) fusion; Ap^R^  (maintained in *E. coli* TB1) |  |
| pEnterprise | Source of the *picard* (Cm^R^) Tn |  |
| pFalcon | Source of the *solo* (Km^R^) Tn |  |
| pGEM-T | PCR cloning vector; Ap^R^ | Promega |
| pUC18-K2 | Source of non-polar *aphA3* cassette; Ap^R^Km^R^ |  |
| pRRC | *C. jejuni* rRNA spacer integration vector; Cm^R^ |  |
| pRRK | *C. jejuni* rRNA spacer integration vector; Km^R^ | J. Ketley |
| pET-32a(+) |  | Novagen |
| pGEMT-*pgp1* | pGEMT ligated to *pgp1* amplified with 1344For and 1344Rev; Ap^R^ | This study |
| pGEMT-*pgp1*Km | pGEMT-*pgp1* with the *pgp1* gene disrupted with the *aphA3* cassette; Ap^R^Km^R^ | This study |
| pGEMT-*1343* | pGEMT ligated to *1343* amplified with 1343-3 and 1343-4; Ap^R^ | This study |
| pGEMT-*1343*Km | pGEMT-*1343* with the *1343* gene disrupted with the *aphA3* cassette; Ap^R^Km^R^ | This study |
| pEF15 | pRRC-1344 coding for 1344 and 173 bp upstream of 1344; Km^R^ | This study |
| pEF20 | pRRK-1344 coding for 1344 and 173 bp upstream of 1344; Km^R^ | This study |
| pEF35R | pRRC-1344-1348 coding for *pgp1* and 3816 bp upstream of *pgp1*; Cm^R^ | This study |
| pEF46 | pET32a-*pgp1*(aa 16-464) with an N-terminal Trx tag and His tag | This study |

Primers used in this study. Restriction sites are underlined.

| **Primer** | **Sequence 5′ to 3′** | | **Restriction Site** | | **Reference** |
| --- | --- | --- | --- | --- | --- |
| ak233 | GCAAGAGTTTTGCTTATGTTAGCAC | |  | |  |
| ak234 | GAAATGGGCAGAGTGTATTCTCCG | |  | |  |
| ak235 | GTGCGGATAATGTTGTTTCTG | |  | |  |
| aphA3-2 | CTATTTTTTGACTTACTGGGGA | |  | | This study |
| cat-2 | GTTTTTTGGATGAATTACAAGA | |  | | This study |
| CEKG2A | GGCCACGCGTCGACTAGTACNNNNNNNNNNAGAG | |  | |  |
| CEKG2B | GGCCACGCGTCGACTAGTACNNNNNNNNNNACGCC | |  | |  |
| CEKG2C | GGCCACGCGTCGACTAGTACNNNNNNNNNNGATAT | |  | |  |
| CEKG4 | GGCCACGCGTCGACTAGTAC | |  | |  |
| Mariner-2 | TCTTGAAGGGAACTATGTTGA | |  | | This study |
| Mariner-3 | AATACTAGCGACGCCATCTA | |  | | This study |
| IR-1 | GGACTTATCAGCCAACCTG | |  | | This study |
| MarOut-3 | CCGGGGACTTATCAGCCAACC | |  | |  |
| 1344-Forw | CAATGCTGCTAATGAAGTTGG | |  | | This study |
| 1344-Rev | AGCATCAACTGCAGCTCTTG | |  | | This study |
| 1344-1 | ATAATCAGGtaCCTTTGGGCT | | *KpnI* | | This study |
| 1344-2 | CAATACTCTTTAGACATGGCAGG | |  | | This study |
| 1344-5 | CAATGCTGCTAgcGAAGTTGG | | *NheI* | | This study |
| 1344-6 | GAACTTTCTATAGCTAGcATAAGATT | | *NheI* | | This study |
| 1344-7 | CACAAGAACTTTCTATAGCTAGGATAAGATT | |  | | This study |
| 1344-8 | GAACTTTCTcgAGCTAGGATAAGATT | | *XhoI* | | This study |
| 1344-14 | TCACTATAGcTAGcGCGACTATGGC | | *NheI* | | This study |
| 1344-15 | TGAAGTCTTGCAaCtAGTTCAGGTAC | | *SpeI* | | This study |
| 1344-18 | cgacaaggccatgggcTTAGATCTAGACTTTAGCGTAGGAGAAAATGG | | *NcoI* | | This study |
| 1348-1 | GCTCATTTTCAaCTAgTTTAGGACTATCTAC | | *SpeI* | | This study |
| 1343-1 | TTCTTTGCTCAGTGGAATTT | |  | | This study |
| 1343-2 | CGCTTAAATTTTCATGCTTT | |  | | This study |
| 1343-3 | CCTGAGTATTTAGAATTTAGCGATGC | |  | | This study |
| 1343-4 | GCTTTGATTGGCTGCGTTG | |  | | This study |
| 1343-5 | GCCTCGCTATGAAGTCTTGC | |  | | This study |
| 1343-6 | gcgtctagaCAAGAGCTGCAGTTGATGCTTAT | | *XbaI* | | This study |
| **Quantitative PCR TaqMan probe and primers** | | | | | |
| gyrA Probe^1^ | | (6FAM)TATGTGTATCAAATTCCCACTTG(MGB-NFQ) | |  | This study |
| gyrA-QRT-Forw | | ATGCTCTTTGCAGTAACCAAAAAA | |  | This study |
| gyrA-QRT-Rev | | GGCCGATTTCACGCACTTTA | |  | This study |
| pgp1 Probe^1^ | | (6FAM)CCTGAAGTTAGTATGGTGATCA(MGB-NFQ) | |  | This study |
| pgp1-QRT-Forw | | AACCGTGCAACGCATTAAAGA | |  | This study |
| pgp1-QRT-Rev | | AAAACCCCAACCATCGTGAA | |  | This study |

^1^ 5′ 6FAM (6-carboxyfluorescein) and 3'MGB-NFQ (minor groove binder/non-fluorescent quencher)

**REFERENCES**

1. Gotz D, Banta A, Beveridge TJ, Rushdi AI, Simoneit BR, et al. (2002) Persephonella marina gen. nov., sp. nov. and Persephonella guaymasensis sp. nov., two novel, thermophilic, hydrogen-oxidizing microaerophiles from deep-sea hydrothermal vents. Int J Syst Evol Microbiol 52: 1349-1359.

2. Sycuro LK, Pincus Z, Gutierrez KD, Biboy J, Stern CA, et al. (2010) Peptidoglycan crosslinking relaxation promotes Helicobacter pylori's helical shape and stomach colonization. Cell 141: 822-833.

3. Glauner B, Holtje JV, Schwarz U (1988) The composition of the murein of Escherichia coli. J Biol Chem 263: 10088-10095.

4. Akerley BJ, Lampe DJ (2002) Analysis of gene function in bacterial pathogens by GAMBIT. Methods Enzymol 358: 100-108.

5. Hendrixson DR, Akerley BJ, DiRita VJ (2001) Transposon mutagenesis of Campylobacter jejuni identifies a bipartite energy taxis system required for motility. Mol Microbiol 40: 214-224.

6. Salama NR, Shepherd B, Falkow S (2004) Global transposon mutagenesis and essential gene analysis of Helicobacter pylori. J Bacteriol 186: 7926-7935.

7. Menard R, Sansonetti PJ, Parsot C (1993) Nonpolar mutagenesis of the ipa genes defines IpaB, IpaC, and IpaD as effectors of Shigella flexneri entry into epithelial cells. J Bacteriol 175: 5899-5906.

8. Karlyshev AV, Wren BW (2005) Development and application of an insertional system for gene delivery and expression in Campylobacter jejuni. Appl Environ Microbiol 71: 4004-4013.

9. Gaynor EC, Wells DH, MacKichan JK, Falkow S (2005) The Campylobacter jejuni stringent response controls specific stress survival and virulence-associated phenotypes. Mol Microbiol 56: 8-27.

10. Korlath JA, Osterholm MT, Judy LA, Forfang JC, Robinson RA (1985) A point-source outbreak of campylobacteriosis associated with consumption of raw milk. J Infect Dis 152: 592-596.

11. McLennan MK, Ringoir DD, Frirdich E, Svensson SL, Wells DH, et al. (2008) Campylobacter jejuni biofilms up-regulated in the absence of the stringent response utilize a calcofluor white-reactive polysaccharide. J Bacteriol 190: 1097-1107.

12. Edwards RA, Keller LH, Schifferli DM (1998) Improved allelic exchange vectors and their use to analyze 987P fimbria gene expression. Gene 207: 149-157.
